# Rapid, Accurate and Reproducible Prediction of the Glass Transition Temperature Using Ensemble-Based Molecular Dynamics Simulation

**DOI:** 10.1021/acs.jctc.4c01364

**Published:** 2025-01-29

**Authors:** James L. Suter, Werner A. Müller, Maxime Vassaux, Alexandros Anastasiou, Martin Simmons, David Tilbrook, Peter V. Coveney

**Affiliations:** † Centre for Computational Science, University College London, 20 Gordon Street, London WC1H 0AJ, U.K.; ‡ Institute of Physics, Université de Rennes, CNRS, IPR - UMR 6251, Rennes 35000, France; § Hexcel Composites, Ickleton Road, Duxford, Cambridge, Cambridgeshire CB22 4QD, U.K.; ∥ Advanced Research Computing Centre, University College London, London WC1E 6BT, U.K.; ⊥ Computational Science Laboratory, Institute for Informatics, Faculty of Science, University of Amsterdam, Amsterdam 1098XH, The Netherlands

## Abstract

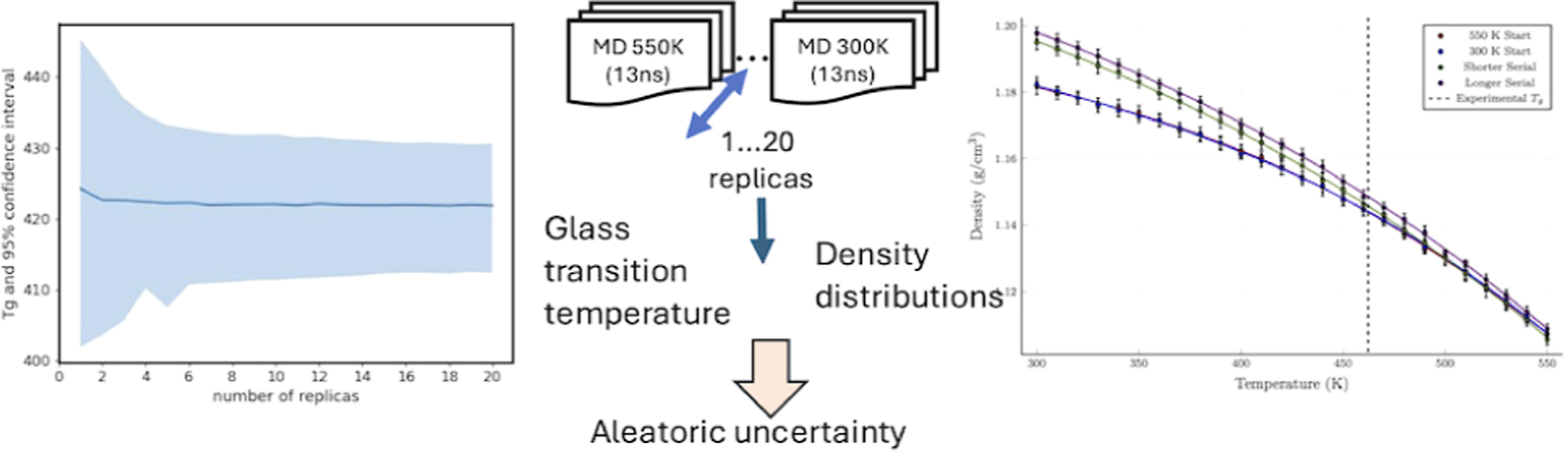

For the computational design of new polymeric materials, accurate methods for determining the glass transition temperature (*T*_g_) are required. We apply an ensemble approach in molecular dynamics (MD) and examine its predictions of *T*_g_ and their associated uncertainty. We separate the uncertainty into the aleatoric contributions arising from dynamical chaos and that due to the computational scenarios chosen to compute *T*_g_. We propose a new scenario for computing *T*_g_, where the density–temperature behavior is computed by running all temperatures concurrently, rather than invoking a sequential approach, thereby significantly reducing wall-clock time from days to several hours without increasing the aleatoric uncertainty. On comparing concurrent and sequential scenarios on six highly cross-linked epoxy resins cured with aromatic amines, we find excellent agreement with our experimentally determined *T*_g_ using dynamical mechanical analysis for both scenarios. The confidence intervals are found to scale as *N*^–0.5^, where *N* is the number of members in the ensemble, implying that ensembles comprised of at least ten replicas are required to predict *T*_g_ using MD with 95% confidence intervals of less than 20 K. The optimal MD simulation protocol is 4 ns of burn-in time followed by 2 ns of production run time.

## 1 Introduction

The glass transition temperature (*T*_g_) is an important property of amorphous materials. It is the temperature at which the material undergoes a transition from a rigid, glassy state to a softer, rubbery or viscous state. Thermoset and thermoplastic polymers are the archetypal kinds of materials that exhibit a glass transition. Below the glass transition temperature, polymer chains are in a rigid, glassy state. The polymer chains are tightly packed and exhibit limited movement, resulting in high stiffness and brittleness. At temperatures above *T*_g_, the polymer chains have increased mobility and slide or rotate more freely. This leads to a decrease in the polymer’s stiffness, making it more rubbery or viscous. The glass transition temperature is a key parameter to consider when designing and using thermoset or thermoplastic materials. It determines the operating temperature range and uses for the material, whether in the glassy state (for example, hard plastics) or rubbery state (for example, rubber elastomers).

Classical molecular dynamics (MD) can be used to estimate *T*_g_ by simulating the change from glassy to rubbery behavior as a function of temperature. In addition, MD can be used to compute *T*_g_ for potential polymeric materials without requiring their time-consuming synthesis and processing, thereby opening up the possibility of using molecular simulation for rational design of high performance materials.

However, it is an unanswered question as to how reliable *T*_g_ predictions from MD are. It is common in the literature to see an estimate of *T*_g_ computed from only a small number of classical MD simulations. In the literature one finds examples of single,^1−3^ three,^4−6^ four,^7^ five,^8−13^ six^14^ and ten^15−17^ independent molecular dynamics simulations of the same system to determine an average predicted glass transition. However, little attention has been addressed to the uncertainty accompanying such computed studies of *T*_g_. Moreover, such calculations inevitably involve sequential simulations, leading to very long wall-clock times to compute *T*_g_.

Without uncertainty quantification (UQ), the results of single or a small number of simulations cannot be considered reliable and reproducible, and we cannot have confidence in our predictions.^18^ In this paper, we apply ensemble-based MD methods, in which a set of replicas are run concurrently, to provide reliable and repeatable statistics, in a similar way to what we have done for the Young’s modulus of epoxy systems^19,20^ and in calculating binding affinities of ligand–protein systems.^19^

Ensemble-based methods provide the correct statistical-mechanical way in which to calculate macroscopic quantities from microscopic dynamics.^21^ Beyond this, the statistics gives us a measure of the error of any prediction we make.^19,21−23^ This can be naturally extended to assess the uncertainty attaching to the model parameters too where ensembles play a central role. This leads into the field of UQ, which has not been addressed extensively within most of the physics and chemistry communities, while ensemble approaches have long been established for domains such as weather forecasting and climate modeling.^24^ We refer the reader to a small number of publications which provide a good introduction to the field and help to clarify the jargon used therein.^20,25−27,60^

By providing a quantification of the error in our predictions, we provide a key measure of the confidence with which we can report modeling results and, in turn, use them as an actionable guide for experimental work.^28^ In addition, ensemble approaches are highly scalable: they can, for example, permit hundreds to thousands of binding affinities to be calculated per day, depending on the computing resources available.^19^

In UQ, we first divide the sources of uncertainty into epistemic and aleatoric components.^19,29^ Epistemic uncertainty, or systematic error, is introduced by inaccuracies such as imperfect design, parametrization and/or analysis of a study, which result in an estimate of a property deviating consistently from its true value.^29^ There is also “scenario uncertainty”, exemplified here by the scenario in terms of which we choose to calculate *T*_g_ from classical MD.^30^

Aleatoric uncertainty, also called stochastic uncertainty or system noise, arises due to the presence of random seeds in an MD code and is caused by the chaotic nature of classical MD, leading to fluctuations around an average value. The extreme sensitivity of classical MD to initial conditions can be quantified using ensemble methods, where replicas (model simulations at the same epistemic parameters and a different random seed initializing the model) are executed concurrently on a large enough computer, and statistical averages from all the simulations are calculated. In the ergodic hierarchy of dynamical systems, those which approach and reach equilibrium must be at least mixing.^22^ Neighboring MD trajectories in such systems, no matter how close they are initially in phase space, diverge exponentially fast with the passage of time.^21^ Therefore, substantially different microstates are generated by the replicas. The key feature of ensemble simulation is the use of ensemble and time averaging.^19,21^

Comparison with experiment for validation is complicated by “model structure uncertainty”; that is, how representative our molecular model is of the experimentally observed system and the processes that occur during the glass transition.^31^ In this paper we consider thermoset polymers, for which we have significant model uncertainty related to the degree of cross-linking within the sample. Quantifying the degree of cross-linking is challenging and highly dependent on processing conditions and technique used. However, it can considerably alter *T*_g_. Relevant to the systems studied in this paper, in the Supporting Information we give examples from the literature illustrating the variation in *T*_g_ values determined experimentally for a representative epoxy resin used in this study, as a function of degree of cross-linking and experimental technique. We find variations in reported *T*_g_ values of between 20 and 30 K. A detailed review of the sources of variation in experimentally determined *T*_g_ can be found in the paper by Hutchinson.^32^

We focus on using ensemble methods to compute the uncertainties arising from aleatoric uncertainty, i.e. the inherent noise in computing *T*_g_ from classical MD and compare it with variation in *T*_g_ originating from different measurement protocols. We have shown in our previous studies that the aleatoric uncertainty is the dominant source of uncertainty in MD simulations, excluding interaction parameters.^19,33^ The motivation for the current paper is the need for best-practices when performing high-throughput simulations, including reporting reproducible predictions. There are numerous parameters associated with the simulations whose effects could be assessed. In this paper, we have restricted ourselves to a few common variables (such as the replicas in an ensemble, data collection times, the method to extract *T*_g_).

We have also made a series of experimental measurements of *T*_g_. Due to the large scenario uncertainty, we validate the scenarios by their ability to produce the same trends or ranking observed experimentally, rather than in terms of their absolute values.

The paper is organized as follows. In Section 2.1 we define the thermoset polymers we have considered for this study. In Section 2.2 we discuss the different scenarios for computing *T*_g_ from classical MD, the various protocols employed to compute *T*_g_ and the associated aleatoric uncertainty for each. In Section 3.1 we validate our computed *T*_g_ through comparison with experiment. In Section 3.2, we examine the convergence with the number of replicas in the ensemble and in Section 3.3, the duration of the classical MD simulations. In Section 3.4, we examine whether the computed *T*_g_ values are normally distributed; we also report the atomic density, from which *T*_g_ is derived. Finally, in Section 4, we provide the conclusions from our work, including recommendations for optimal ensemble setup and scenario parameters for the reliable determination of *T*_g_.

## 2 Computational Scenarios and Protocols

### 2.1 Polymer Systems

**Figure 1:**
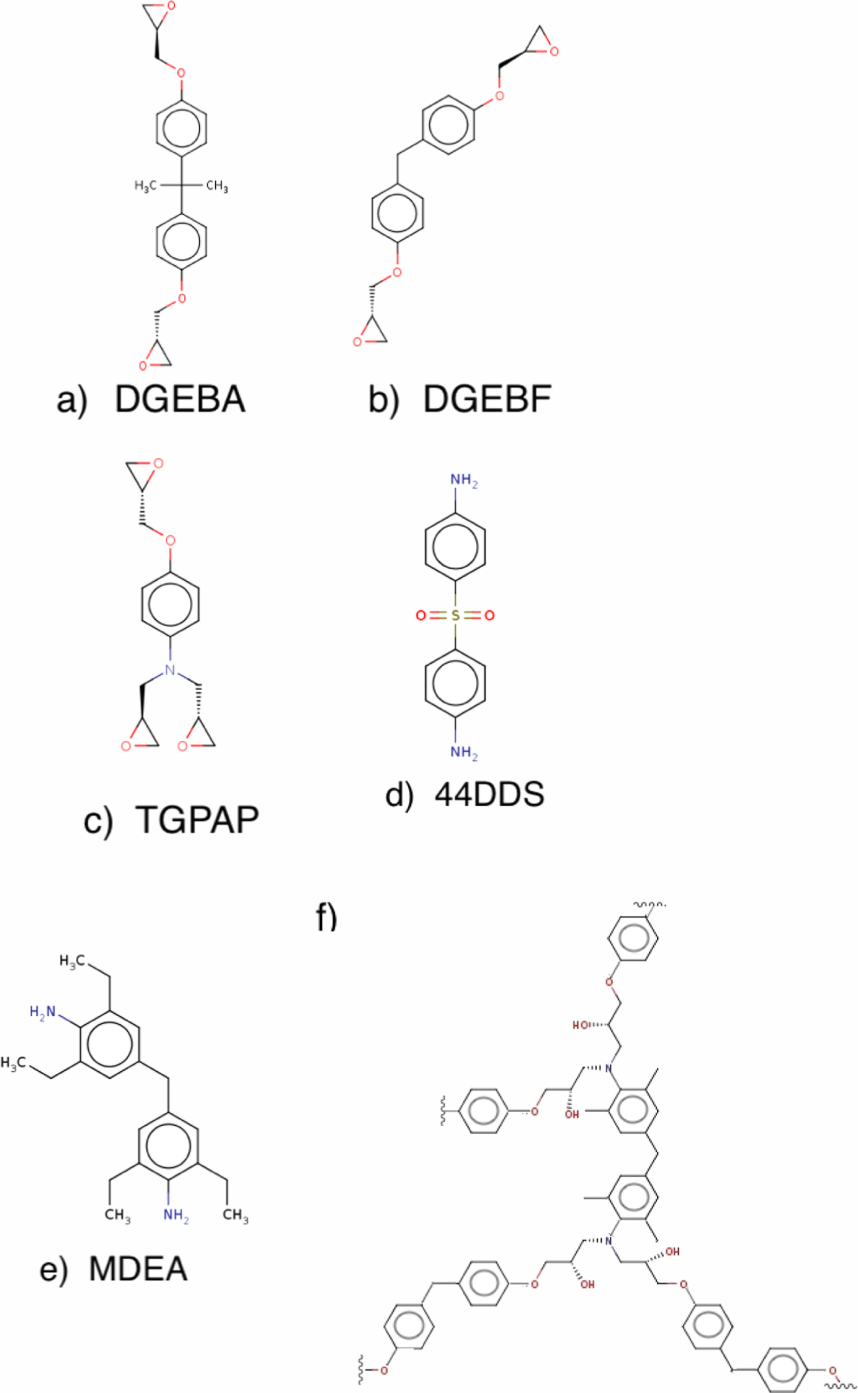
Different amine-cured epoxies used in this study. The epoxy molecules are (a) DGEBA, (b) DGEBF and (c) TGPAP, and the curatives are (d) 44DDS and (e) MDEA. The epoxy molecules and curatives react to create a cross-linked structure. (f) The cross-linked structure of a selected part of the DGEBF–MDEA epoxy resin (model **IV**) illustrating the functional groups created during the ring-opening cross-linking between the epoxy and curative precursors.

We choose industrially important cross-linked epoxy resins to examine the uncertainty associated with computing *T*_g_ using MD (see Figure 1). These molecules are widely used in paints, coatings, adhesives and structural components in the aerospace industry.^34,35^

First, we consider the common and commercially available epoxy resin, diglycidyl ether of bisphenol A (DGEBA) (Figure 1a) cured with aromatic diamines 4,4′-diaminodiphenyl sulphone (44DDS) (Figure 1d) and 4,4′-methylene-bis(2,6-diethylaniline) (MDEA) (Figure 1e), which are referred to as models **I** and **II** in the rest of this paper respectively (see Table 1).

**Table 1:** Resin Models Used in This Study^a^

model	epoxy	curative	experimental *T*_g_ (K)	room temperature experimental density (g/cm^3^)
**I**	DEGBA	44DDS	462.3 ± 4.9	1.22
**II**		MDEA	448.8 ± 11.9	1.14
**III**	DEGBF	44DDS	448.9 ± 5.7	1.28
**IV**		MDEA	416.6 ± 4.3	1.15
**V**	TGPAP	44DDS	538.1 ± 2.9	1.32
**VI**		MDEA	487.3 ± 2.4	1.16

a The cross-linked systems are created by the MedeA thermoset builder with a cross-linked ratio of 95%. The experimental *T*_g_ has been determined by DMA (see Computational Methods for details).

Second, we consider the diglycidyl ether of bisphenol F (DGEBF) (Figure 1b) as the epoxy resin, which differs from DGEBA by possessing a central methylene group linking the aryl groups, rather than an isopropyl group. DGEBF cured with 44DDS and MDEA are referred to as models **III** and **IV** respectively. The high glass transition of the resultant cured epoxy resins of DGEBA and DGEBF (>200 °C) is thought to be due to the aromatic chain backbone, which provides stiffness and strength to the resin. We have chosen to compare DGEBA and DGEBF as the only difference in their chemical structures is one group on the aromatic backbone so that the resulting *T*_g_ values are expected to be similar. These resins therefore provide a good reference data set to establish whether we can distinguish between the two epoxies with our predictions and if these are also in agreement with the experimental *T*_g_ values.

Third, we consider the trifunctional epoxy triglycidyl-para-amino-phenol (TGPAP) (Figure 1c), which contains three epoxy groups per monomer unit. The TGPAP resins provide a good test of the effect of extra cross-linking per monomer unit. TGPAP cured with 44DDS and MDEA are referred to as models **V** and **VI** respectively. The chemical structures of epoxies and curatives are shown in Figure 1. Table 1 lists the six cross-linked systems used in this study as well as their experimental *T*_g_ values. All cross-linked structures are built with a cross-linking ratio of 95% (see Computational Methods for more details).

We can qualitatively relate the experimental glass transition temperatures (*T*_g_) shown in Table 1 to the structural features of the epoxy resins and curing agents by examining how these features restrict polymer chain mobility. Structural elements that impose greater constraints on molecular motion typically lead to higher *T*_g_.

The higher *T*_g_ observed in epoxy resins derived from DGEBA compared to DGEBF can be attributed to the isopropyl groups in the chain segment of DGEBA. These groups hinder large-scale conformational changes, thereby reducing chain flexibility and increasing *T*_g_. In contrast, the higher degree of cross-linking per precursor molecule in TGPAP (which contains three epoxy groups, versus two in DGEBA and DGEBF) results in even higher *T*_g_ for epoxy resins formulated with TGPAP precursors. The chemical structure of the curing agents also plays a crucial role in determining *T*_g_. 44DDS features a rigid sulfone (–SO_2_–) linkage between aromatic groups, contributing to a stiff and less flexible network, which raises *T*_g_. In contrast, MDEA’s methylene linkage and bulky ethyl substituents introduce greater flexibility into the polymer matrix, resulting in lower *T*_g_ values for epoxy resins cured with MDEA compared to those cured with 44DDS.

### 2.2 Scenarios for the Estimation of the Glass Transition Temperature

Computing the glass transition temperature involves two steps: computing the density as a function of temperature and subsequently using the density–temperature curve to determine *T*_g_. While this procedure is the most widely used method to determine *T*_g_(17) from classical MD, there are alternative methods using dynamical properties such as mean-squared displacements (MSD) or segmental relaxation times. However, in automated high-throughput scenarios, we wish to compute *T*_g_ using easily accessible bulk properties such as density, or other thermophysical properties, rather than dynamical properties that are more involved to extract. Other authors have sought to make better predictions by combining molecular dynamics with data driven analysis, by employing statistical methods to identify such correlations, be they structural or dynamical. Such machine learning methods have enjoyed limited qualitative success in this domain when applied to very simple polymeric systems.^36−38^ None of these approaches involve any form of uncertainty quantification. For complex chemically specific polymer networks such correlations are unlikely to be identifiable let alone predictive in a quantitative sense; whereas in this study, we make quantitative predictions of the glass transition temperature with controlled error estimates.

In the Supporting Information, we find the *T*_g_ values using segmental relaxation times and MSD to be broadly similar to those using the density–temperature behavior, although the various fitting steps when using the dynamical properties (outlined in the Supporting Information) introduce significant statistical error. We have therefore focused on using the density–temperature behavior in this study. However, the analysis does provide some insight into the processes that occur during the glass transition. Analysis of phenyl carbon–hydrogen bonds shows a clear deviation from Arrhenius behavior, indicating the glass transition, while carbon–oxygen bonds remain consistent with Arrhenius behavior. This suggests that the motion of phenyl rings is the dominant segmental motion associated with *T*_g_. We study two different computational scenarios to compute the density–temperature behavior, which differ in the amount of concurrent simulations required. Here we list the different computational protocols used for each scenario.

1. Stepwise/sequential scenario: the system is run for 13 ns at a high temperature (550 K for models **I–IV** and 650 K for models **V** and **VI**) and subsequently cooled down in steps of 10 K of duration of either 0.5 or 2 ns, i.e. the MD simulations at each temperature are computed sequentially down to a final temperature of 300 K for models **I–IV** and 400 K for models **V** and **VI**. These are cooling rates commonly used for *T*_g_ MD studies (for example, ref (17)). The duration of the MD simulations is sufficient for the densities to reach a steady state on the time scale of MD, as determined by automated methods of detecting equilibration/meta-stability^39−41^ (see Supporting Information Section 4 for more details). Therefore, the starting configuration for a given temperature is the final configuration of the next-highest temperature. Twenty sequential stepwise runs were performed concurrently to create an ensemble of 20 stepwise replicas.
2. Parallel/concurrent scenario: the system is simulated at a high temperature for 13 ns as per the stepwise scenario. The final snapshots are then quenched to create the starting structures for all required temperatures. Unlike the stepwise protocol, all MD simulations at different temperatures are run concurrently. The duration of the MD simulations is 13 ns, which is sufficient to reach a steady state on the time scale of MD, as determined by automated methods of detecting equilibration/meta-stability^39−41^ (see Supporting Information Section 4). Twenty parallel runs are performed concurrently to create an ensemble of 20 parallel replicas. In total, therefore, a maximum of 520 MD simulations can be run concurrently (26 temperature points ×20 replicas) for each system.

As an additional protocol, provided a supercomputer of sufficient size is available, the system is simulated at a low temperature for 13 ns, with the final snapshot used as the starting structure for each temperature. This was used to test whether further simulation at low temperatures provides a better description of the glassy regime.

On a point of terminology, for the parallel scenario, using the configurations from high temperatures will be referred to as the *parallel* protocol, while that with a 13 ns simulation step at low temperatures will be referred to as the *parallel_cold* protocol. Regarding the stepwise scenario, we refer to that with the 0.5 ns duration MD simulation per 10 K step as *stepwise_0.5ns* protocol; *stepwise_2ns* protocol denotes the 2 ns duration equivalent.

The wall-clock time for running temperature points in parallel is far less than that of the stepwise protocols, where the temperature points must be computed sequentially. For the systems under consideration here, the stepwise_2ns protocol took an average of 2 days using 128 cores per replica for the ARCHER2 supercomputer (http://www.archer2.ac.uk/), compared to 6 h for the parallel protocol.

In extensive earlier research, we have shown that ensemble MD, with fixed epistemic parameters and initial atomic velocities of each replica drawn from a random seed-based Maxwell–Boltzmann distribution, quantifies the aleatoric uncertainty.^19,20,22,23^ It should be pointed out that exactly this description is what defines the concept of “ensembles” in statistical mechanics and allows one to calculate macroscopic properties from microscopic states. Therefore, only the random seed for the initial atomic velocities drawn from a Maxwell–Boltzmann distribution is different between the replicas within each ensemble. In addition, in our previous studies, we found that the exact connectivity of monomer units of epoxy resins was inconsequential in determining the Young’s modulus, provided that the same cross-link density was achieved within all samples; the variance in predicting the Young’s modulus is solely due to the use of different initial velocities, which can be attributed to microscopic states with different probabilities being sampled by each replica due to their extreme sensitivity to initial conditions.^19,20^

Conventionally, *T*_g_ is determined from the density–temperature curve by the identification of two linear lines, one representing the asymptotic behavior at low temperature in the glassy regime and the other the asymptotic behavior at high temperature in the rubbery regime. The glass transition lies between these two asymptotic regimes and is associated with the intersection of the two asymptotic lines. Identification of the asymptotic regimes is traditionally done by eye, which can be problematic when the transition between regimes is gradual, as is often the case with MD simulations. Therefore, we investigate two methods commonly used to determine *T*_g_ from the density–temperature behavior which do not need user intervention and hence can be deployed for automated determination of *T*_g_. The method of Patrone et al.^42^ fits the entire curve to a hyperbolic function. Denoting ρ to be the average density at a temperature point *T*,
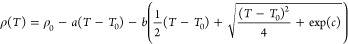
1where *T*_0_, ρ_0_, *a*, *b*, *c* are positive real constants. The glass transition temperature is given by *T*_0_ which represents the center of the hyperbola. We refer the determination of *T*_g_ using eq 1 as the “Patrone fitting method” in the rest of the paper.

The method of Lin et al.^43^ computes a linear fit to the whole curve and subsequently divides up the curve into regions above and below the glass transition depending on their *R*^2^ values without user interaction. A linear fit is then computed for the low- and high-temperature regions. *T*_g_ is then calculated from the intersection of the linear fits to the low- and high-temperature regions. We refer to the bilinear fit of Lin et al.^43^ in the rest of the paper as the “Lin fitting method”. Note that other methods for automating the density–temperature to a bilinear fit have recently been published.^44,45^

To compute the density–temperature behavior using the parallel protocols, we calculate the mean density across the ensemble at each temperature point. To determine the aleatoric uncertainty of the predicted *T*_g_, bootstrap with replacement is performed. 1000 resamples from the distribution of densities of the original 20 replicas are taken, at random with replacement, at each temperature. We are therefore using bootstrapping to probe the variability in ensemble-averaged densities at each temperature. We vary the size of the bootstrap sample to examine the effect of ensemble size. Repeating this procedure at each temperature point builds 1000 average density–temperature curves; *T*_g_ is computed for each curve using the Patrone and Lin fitting methods described above. From the 1000 density–temperature curves we can extract the mean *T*_g_, its standard deviation and 95% confidence intervals along with statistical properties of the distribution.

**Figure 2:**
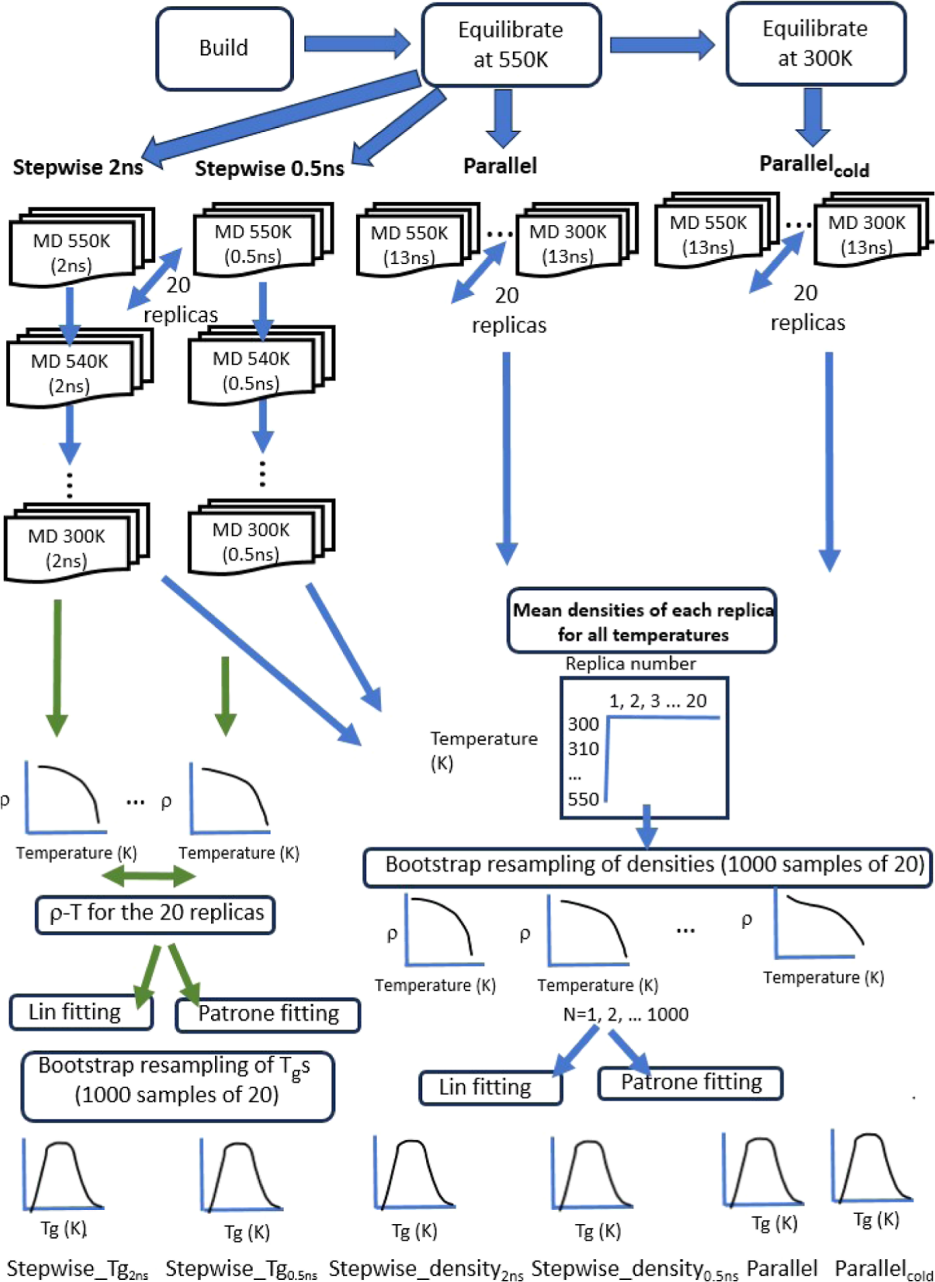
Workflows for computing the glass transition. After building the system using the MedeA thermoset builder and 13 ns simulation at 550 K (650 K for models **V** and **VI**), we ran ensemble MD using the stepwise_2ns, stepwise_0.5ns, parallel and parallel_cold protocols. Twenty replicas are run for each protocol as described in Section 2. The resulting ensemble of 20 densities at each temperature are sampled by bootstrapping the mean of the density at each temperature to create 1000 density–temperature curves, which are subsequently fitted using the methods of Lin and Patrone to create *T*_g_ distributions (blue arrows). For the stepwise protocols, we can also fit the density–temperature curve for each replica to create 20 *T*_g_s. Bootstrapping the mean of the 20 *T*_g_s creates the distributions for the stepwise_Tg protocols (green arrows).

For the stepwise protocol, we have two choices for determining *T*_g_ from fitting procedures. We can compute the average density across the replicas at each temperature point, resampled using the bootstrap method, and from this compute *T*_g_ using the fitting procedures, as per the parallel protocol. We refer to these protocols as *stepwise_density* here. Alternatively, we can compute *T*_g_ for each replica; resampling the *T*_g_ ensemble using the bootstrap method, which we refer to as *stepwise_Tg*. For the parallel protocol, we compute the average densities for each replica using the final 1 ns for each replica (i.e., from 12 to 13 ns). For the stepwise_0.5ns protocol, we average over the final 0.4 ns (0.1–0.5 ns), while for stepwise_2ns we average over the final 1 ns (1–2 ns). The workflow used to compute our *T*_g_ predictions is shown in Figure 2.

The nomenclature of the protocols, and their decomposition into their methods for computing the density–temperature behavior and fitting procedure, is summarized in Table 2.

**Table 2:** Different Protocols We Have Used in This Study to Compute *T*_g_ from Classical MD Simulation^a^

protocol	length of simulation (ns)	density collection (ns)	resampling	fitting procedure
parallel	13	12–13	density	Patrone/Lin
parallel_cold	13	12–23	density	Patrone/Lin
stepwise_density_2ns	2	1–2	density	Patrone/Lin
stepwise_Tg_2ns	2	1–2	*T*_g_	Patrone/Lin
stepwise_density_0.5ns	0.5	0.1–0.4	density	Patrone/Lin
stepwise_Tg_0.5ns	0.5	0.1–0.4	*T*_g_	Patrone/Lin

a Each protocol is composed of a computational protocol for computing the density–temperature behaviour and a fitting procedure to extract *T*_g_.

We further examine the dependence of *T*_g_ on the length of the MD simulations before density collection starts and on the latter’s duration in Section 3.

## 3 Results and Discussion

### 3.1 *T*_g_ Validation against Experiment

**Figure 3:**
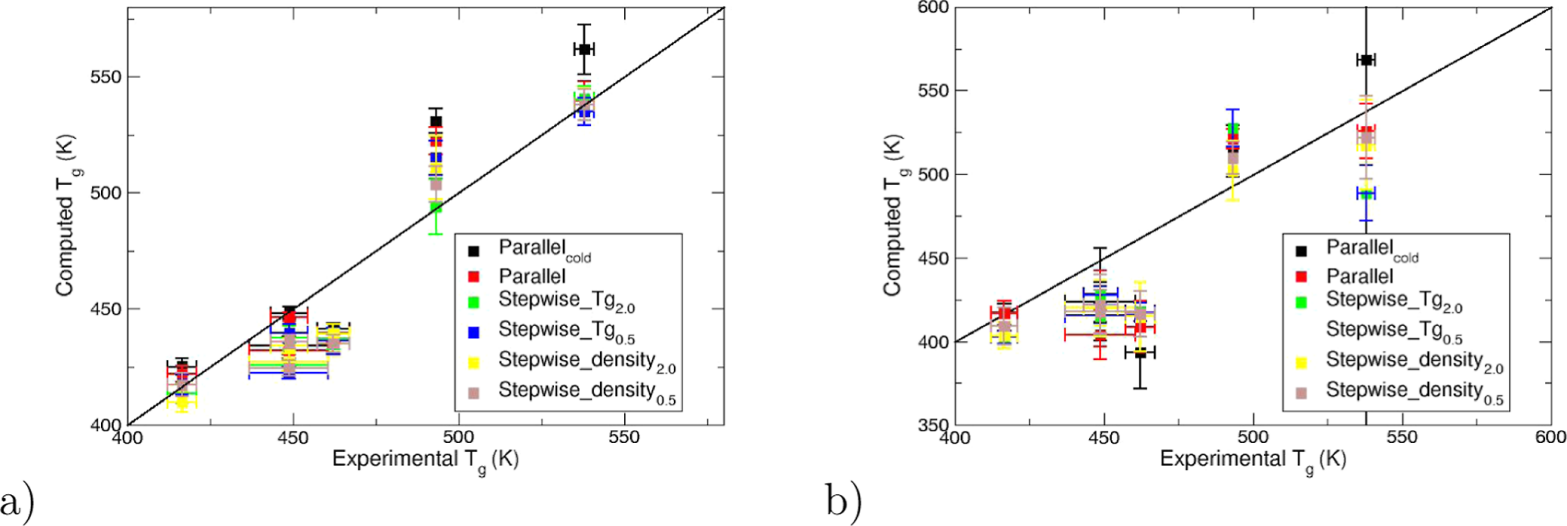
Comparison of calculated glass transition temperatures for the cross-linked epoxy–resins created through the reaction of epoxy monomers and curatives shown in Figure 1. The black line shows an ideal *y* = *x* regression. The standard errors are calculated using a bootstrapping method to create density–temperature curves which are subsequently fitted to determine *T*_g_. Error bars are one standard deviation. (a) The fitting method of Patrone et al. is used.^42^ (b) The fitting method of Lin et al. is used.^43^

In Figure 3, we show a comparison of our predictions for the various scenarios and their associated uncertainties with our experimental data. We report the errors compared to experiment for the Patrone and Lin fitting methods in Table 4. The full list of *T*_g_ predictions is given in Table 3 for the Patrone and Lin fitting methods. Excellent agreement is observed between calculations for all scenarios using the fitting method of Patrone. Compared to the fitting method of Patrone, the errors compared to experiment are approximately twice as large when using Lin’s method (Table 4). The fitting method of Lin et al.^43^ is computed through the intersection of the glassy and rubbery asymptotic lines. Therefore, a much smaller number of data points are used compared to the whole curve being invoked in the fitting method of Patrone et al. and larger errors are reported as a result.

**Table 3:** Comparison of *T*_g_ Predictions Using the Fitting Method of (a) Patrone and (b) Lin with Experiment Values

(a) Patrone method							
resin	experiment (K)	parallel	parallel_cold	stepwise_density_2ns	stepwise_Tg_2ns	stepwise_density_0.5ns	stepwise_Tg_0.5ns
**I**	462 ± 5	439 ± 4	441 ± 3	439 ± 4	437 ± 4	435 ± 4	436 ± 6
**II**	449 ± 12	432 ± 4	434 ± 4	427 ± 4	426 ± 3	425 ± 3	422 ± 3
**III**	449 ± 6	447 ± 2	448 ± 3	434 ± 3	437 ± 5	436 ± 3	439 ± 4
**IV**	417 ± 4	422 ± 3	425 ± 4	410 ± 5	414 ± 4	417 ± 5	417 ± 5
**V**	538 ± 3	540 ± 8	562 ± 11	538 ± 7	540 ± 6	538 ± 7	535 ± 6
**VI**	487 ± 3	522 ± 6	531 ± 5	511 ± 14	494 ± 12	504 ± 8	515 ± 7
(b) Lin Method							
**I**	462 ± 5	409 ± 16	393 ± 21	415 ± 21	417 ± 6	416 ± 14	424 ± 6
**II**	449 ± 12	404 ± 15	423 ± 12	420 ± 11	415 ± 5	418 ± 13	419 ± 4
**III**	449 ± 6	420 ± 23	428 ± 28	420 ± 17	428 ± 5	422 ± 18	422 ± 5
**IV**	417 ± 4	417 ± 7	417 ± 6	403 ± 8	403 ± 4	409 ± 11	412 ± 5
**V**	538 ± 3	526 ± 17	568 ± 237	517 ± 27	489 ± 17	522 ± 25	539 ± 8
**VI**	487 ± 3	521 ± 6	514 ± 15	502 ± 18	528 ± 11	510 ± 10	515 ± 10

**Table 4:** Errors for Each Simulation Protocol Compared to Experiment

	Patrone fitting			Lin fitting		
protocol	mean abs. error	RMSE	mean signed error	mean abs. error	RMSE	mean signed error
parallel_cold	17.7	21.2	5.6	27.8	34.6	–10.6
parallel	13.0	16.8	–0.9	28.0	33.4	–18.6
stepwise_density_2ns	14.0	16.2	–8.15	26.5	29.6	–24.1
stepwise_density_0.5ns	13.7	16.6	–10.1	25.5	31.8	–21.7
stepwise_Tg_2ns	11.0	14.9	–10.0	32.9	35.1	–21.4
stepwise_Tg_0.5ns	14.9	17.4	–6.9	19.6	22.9	–16.3

For the Patrone fitting method, the mean absolute errors (MAE) and root-mean-square errors (RMSE) are consistent across all scenarios (between 11.0 and 17.7 K for MAE and 14.9 and 21.2 K for RMSE). However, the RMSE values are greater than the standard deviation in the experimental *T*_g_ values. While the mean signed error (MSE) is negative for the stepwise scenarios, it is very small for the parallel scenarios. This indicates that the stepwise scenarios consistently underestimate *T*_g_ for these epoxy resins.

While we see excellent agreement with our experimental results, as stated in the Introduction, we need to evaluate these results in the context of model uncertainty (i.e., whether our models are correctly representing the experimental samples) and experimental scenario uncertainty (i.e., the experimental procedures and protocols). One source of model uncertainty is the time scale of simulation: cooling in MD simulations is typically performed at a rate of 0.1–0.2 ns/K, compared to seconds or minutes/K in experiment. MD is therefore commonly assumed to overestimate *T*_g_(17) by invoking the William-Landel–Ferry (WLF) model, which suggests that each order of magnitude higher in time equates to an approximately 3 K increase in *T*_g_ when using universal parameters. This would lead to an expected overprediction in *T*_g_ of approximately 40 K compared to experimental *T*_g_. The parameters in the WLF model are related to the material’s free volume and thermal expansion, which are significantly reduced for highly cross-linked thermoset polymers. As a consequence, the expected shift to higher *T*_g_ for MD is expected to be smaller. Molecular dynamics studies of related epoxy systems (DGEBA and ethylene diamine (EDA)), have also found the predicted *T*_g_ to be approximately the same as experiment,^46^ indicating that the WLF model cannot be relied on for highly cross-linked systems.

While there is substantial model and experimental uncertainty in the values for *T*_g_, we expect the trends between compounds to be a more reliable means of validation, as the same experimental scenario is used for each sample. In Table 5 we show that each of the protocols has high Spearman and Pearson correlation coefficients, confirming that each scenario correctly predicts the same trend as experiments.

**Table 5:** Correlation Coefficients for the Different Protocols Comparing Computed *T*_g_ with Experiment

	Patrone fitting				Lin fitting			
scenario	Spearman correlation	Spearman *p*-value	Pearson correlation	Pearson *p*-value	Spearman correlation	Spearman *p*-value	Pearson correlation	Pearson *p*-value
parallel_cold	0.94	0.005	0.95	0.004	0.66	0.156	0.88	0.020
parallel	0.94	0.005	0.94	0.005	0.71	0.11	0.86	0.03
stepwise_density_2ns	1.00	0.000	0.96	0.002	0.77	0.072	0.92	0.008
stepwise_density_0.5ns	0.94	0.005	0.95	0.004	0.66	0.156	0.87	0.025
stepwise_Tg_2ns	0.94	0.005	0.98	0.001	0.89	0.019	0.79	0.060
stepwise_Tg_0.5ns	0.89	0.019	0.93	0.007	0.94	0.005	0.94	0.005

This shows the real-world applicability of ensemble MD as it can differentiate the glass transition temperature of related chemical systems. We find especially high correlation coefficients for the stepwise and parallel_cold scenarios. The correlation coefficients are much higher when using the Patrone method compared to the Lin method (Table 5), therefore in the rest of the paper our analysis concentrates on scenarios using Patrone’s method. Examining the predictions for individual systems (Table 3) using the Patrone fitting method, we find that all protocols correctly predict a decrease in *T*_g_ when the system is changed from model **II** to **IV** (experimental difference ≈32 K). The smaller change in *T*_g_ from model **I** to **III** (≈13 K) is less reproducible, however, with most protocols predicting the glass transitions being within error of each other. We can therefore infer that 13 K is below the resolution of ensemble MD to distinguish between different models.

### 3.2 Dependence of *T*_g_ on the Size the Ensemble

**Figure 4:**
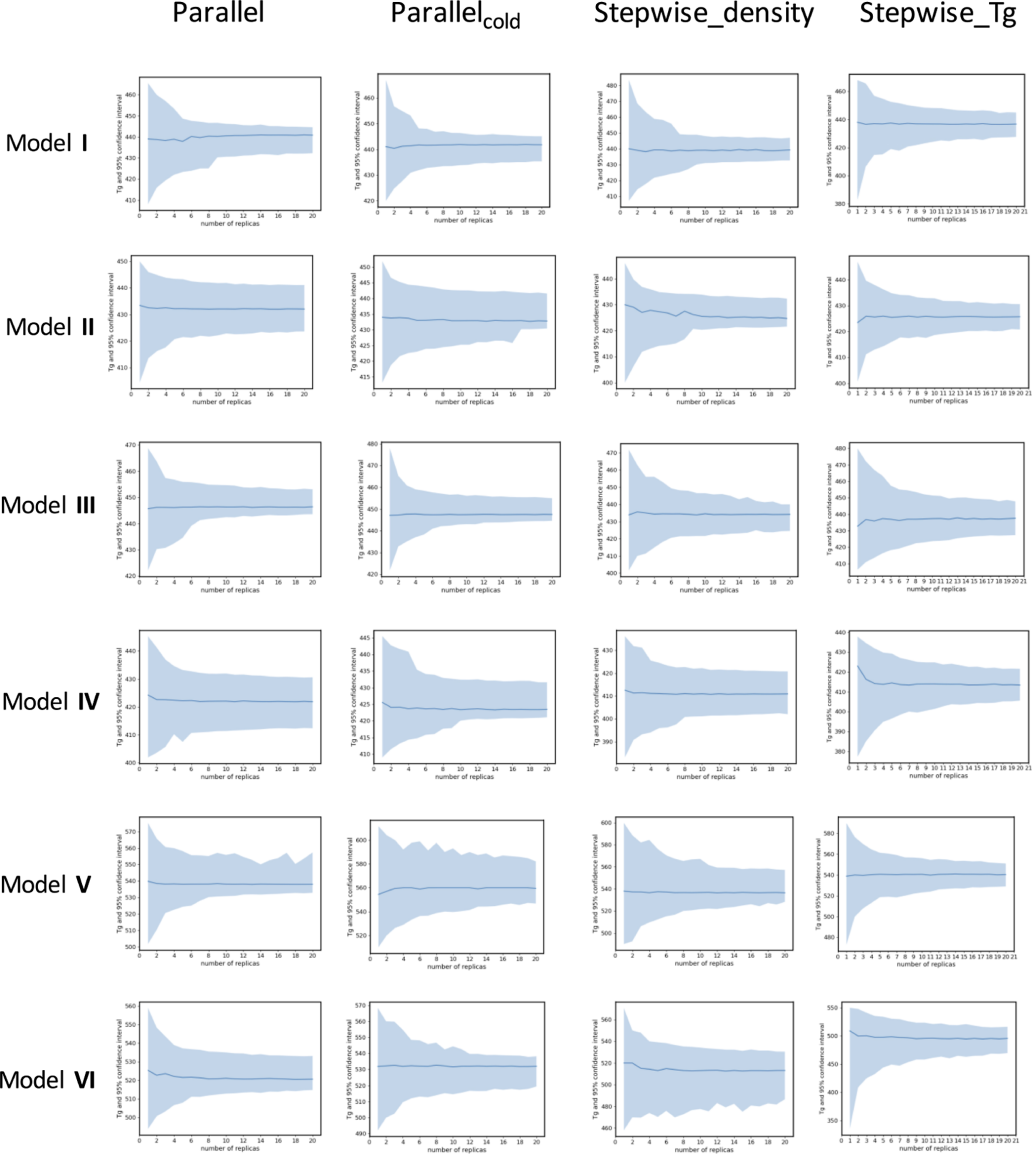
50% percentile values (blue lines) and 95% confidence intervals (blue shaded area) for *T*_g_ predictions using bootstrapping for all models as a function of the size of the ensemble, using the fitting method of Patrone.

Figure 4 shows the predicted *T*_g_ 50% percentile and the 95% confidence intervals (the difference between the 2.5% and 97.5% percentiles) as a function of number of replicas within the ensemble for all models using the Patrone fitting method. The 95% confidence interval is an estimate of the aleatoric uncertainty in our predictions of *T*_g_ as a function of ensemble size.

**Figure 5:**
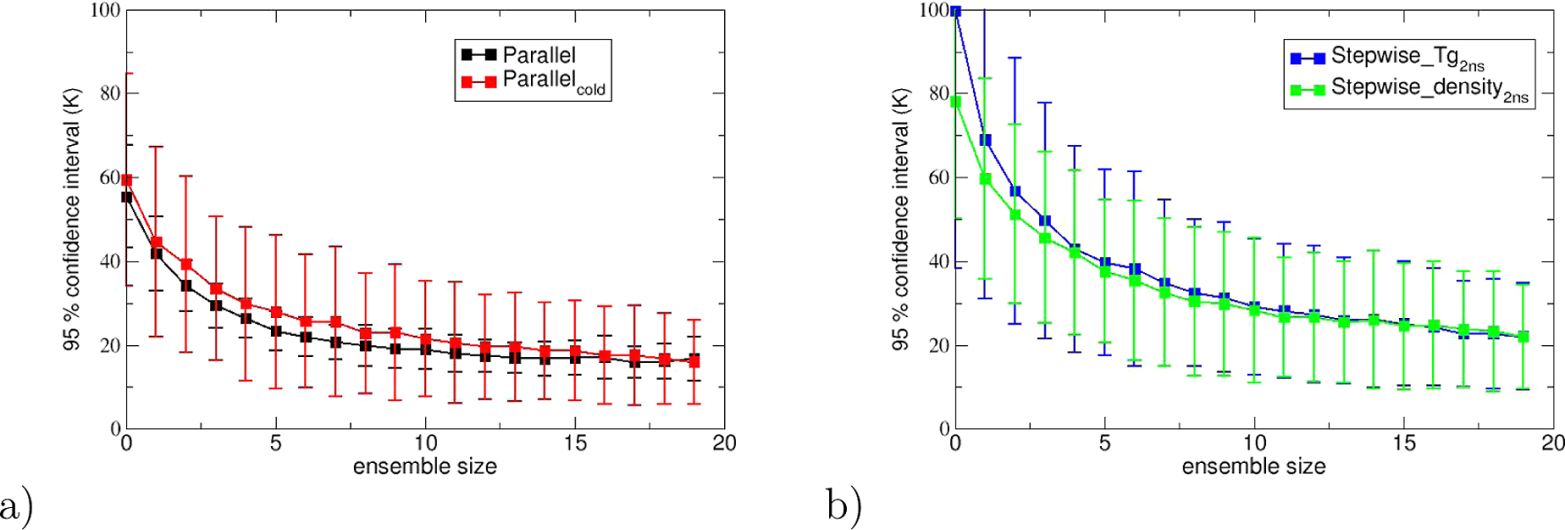
95% confidence intervals for the different protocols as a function of number of replicas in the ensemble, averaged over all cross-linked resin systems. (a) For the parallel and parallel_cold protocols and (b) for the stepwise_Tg_2ns and stepwise_density_2ns protocols.

Figure 4 shows that for all scenarios, the confidence intervals decrease rapidly with the number of replicas in the ensemble. In Figure 5 the dependence of the 95% confidence interval for the different protocols is plotted, averaged over all the resin models in our study. The parallel protocol has a confidence interval of under 20 K at ten replicas, with a higher confidence interval for the stepwise scenarios (≈30 K for stepwise_Tg_2ns and stepwise_density_2ns at ten replicas respectively). The 95% confidence interval with a single replica and for 20 replicas in the ensemble is given in Table 6 for each scenario averaged over all systems, illustrating the very large decrease with *N*. For example, a single replica has 95% confidence interval of 59.42 K for the parallel_cold scenario with the Patrone fitting method, which decreases to 16.0 K when there are 20 replicas in the ensemble. Fitting the 95% confidence interval versus ensemble size we find it is proportional to 1/*N*^0.5^, where *N* is the number of replicas in the ensemble (Table 6). The optimal number of replicas in an ensemble requires a criterion to be chosen by the user, such as the change in 95% confidence interval between *N* and *N* + 1 replicas. In a trade off with the computational cost of the simulations, a sensible cutoff to select is 10 replicas, for which the addition of an extra replica reduces the confidence interval by less than 1 K on average.

**Table 6:** Dependence of 95% Confidence Interval with Ensemble Size for Different Protocols, Averaged over the Different Systems in This Study Using a Single Replica and 20 Replicas, Using the Patrone Fitting Method^a^

protocol	average scaling	average 95% confidence interval (K)	
		1 replica	20 replicas
parallel_cold	–0.45	59.42	16.00
parallel	–0.42	55.39	16.77
stepwise_Tg_2ns	–0.49	99.77	22.10
stepwise_Tg_0.5ns	–0.51	97.93	19.64
stepwise_density_2ns	–0.45	78.14	22.02
stepwise_density_0.5ns	–0.46	77.90	18.78

a The scaling relationship with *N* is also shown, averaged over all systems. We find approximately 1/*N*^0.5^ scaling.

### 3.3 Dependence of *T*_g_ on Duration of MD Simulation of Each Simulation

**Figure 6:**
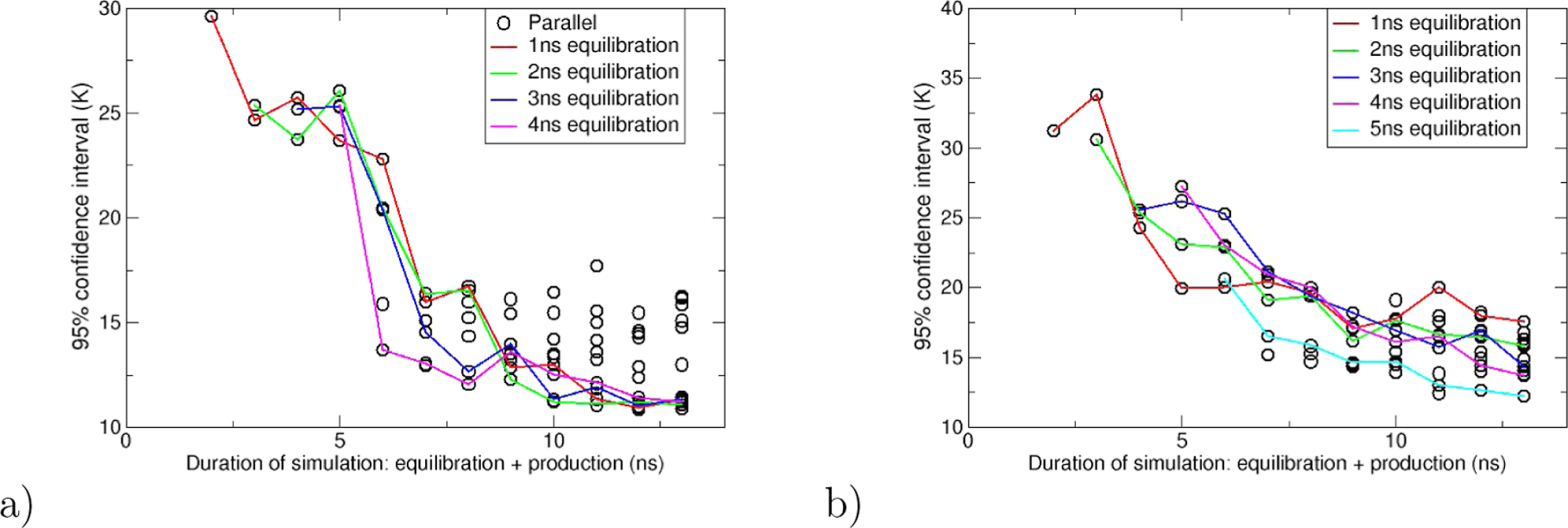
(a) The relationship between the 95% confidence interval and the duration of MD simulation averaged over all models for (a) parallel scenario and (b) parallel_cold protocols. The lines connect the points with the same equilibration/“burn-in” time (red = 1 ns, green = 2 ns, blue = 3 ns, pink = 4 ns, cyan = 5 ns).

Within the parallel scenarios, all MD simulations at different temperatures are run concurrently. There is no cooling rate, unlike the stepwise scenarios, as each simulation is quenched to the required temperature at the start of the MD simulation. However, as all simulations are run concurrently, we can run the individual simulations for longer than in the stepwise scenarios, while still having significantly shorter overall wall-clock time. Therefore, it is necessary to analyze the dependence of the predicted *T*_g_ predictions on the duration of the MD simulation and the time frame used to compute the average density of the system. We can think of the start of density collection as the end of “equilibration” or “burn-in” time. Figure 6 shows the change in 95% confidence interval as a function of total MD simulation time (up to 13 ns) averaged over all resin models for 20 replicas, where the lines connect values with the same burn-in time. Figure 6 shows that there is very little decrease in the 95% confidence intervals when running the MD simulation for longer than 6 ns for the parallel protocol and 7 ns for the parallel_cold protocol. In Supporting Information Section 9, we show that all the predicted *T*_g_ are within the 95% confidence interval with this length of MD simulation. Figure 6 shows that there is no trend suggesting that longer burn-in times than 4 and 2 ns for the parallel and parallel_cold protocols respectively lead to a lowering of the 95% confidence interval. Therefore, we conclude that approximately 4 ns of burn-in time and 2 ns of data collection are sufficient for the parallel protocol and 2 ns of burn-in time and 5 ns of data collection are sufficient for the parallel_cold protocol. It should be noted that this time frame of data collection is similar to the protocols found to be optimal for calculating binding affinities.^18^ In Supporting Information Section 4, we compare these results to using densities determined from automated methods of detecting equilibration (defined as stability on the time scale of the MD simulation),^39−41^ and we observe that the predicted *T*_g_ values are within 95% confidence intervals using the burn-in and data collection times detailed above.

### 3.4 *T*_g_ and Density Distributions

It is important to determine whether our predictions of *T*_g_ and the underlying density distributions are normally distributed.^18^

**Figure 7:**
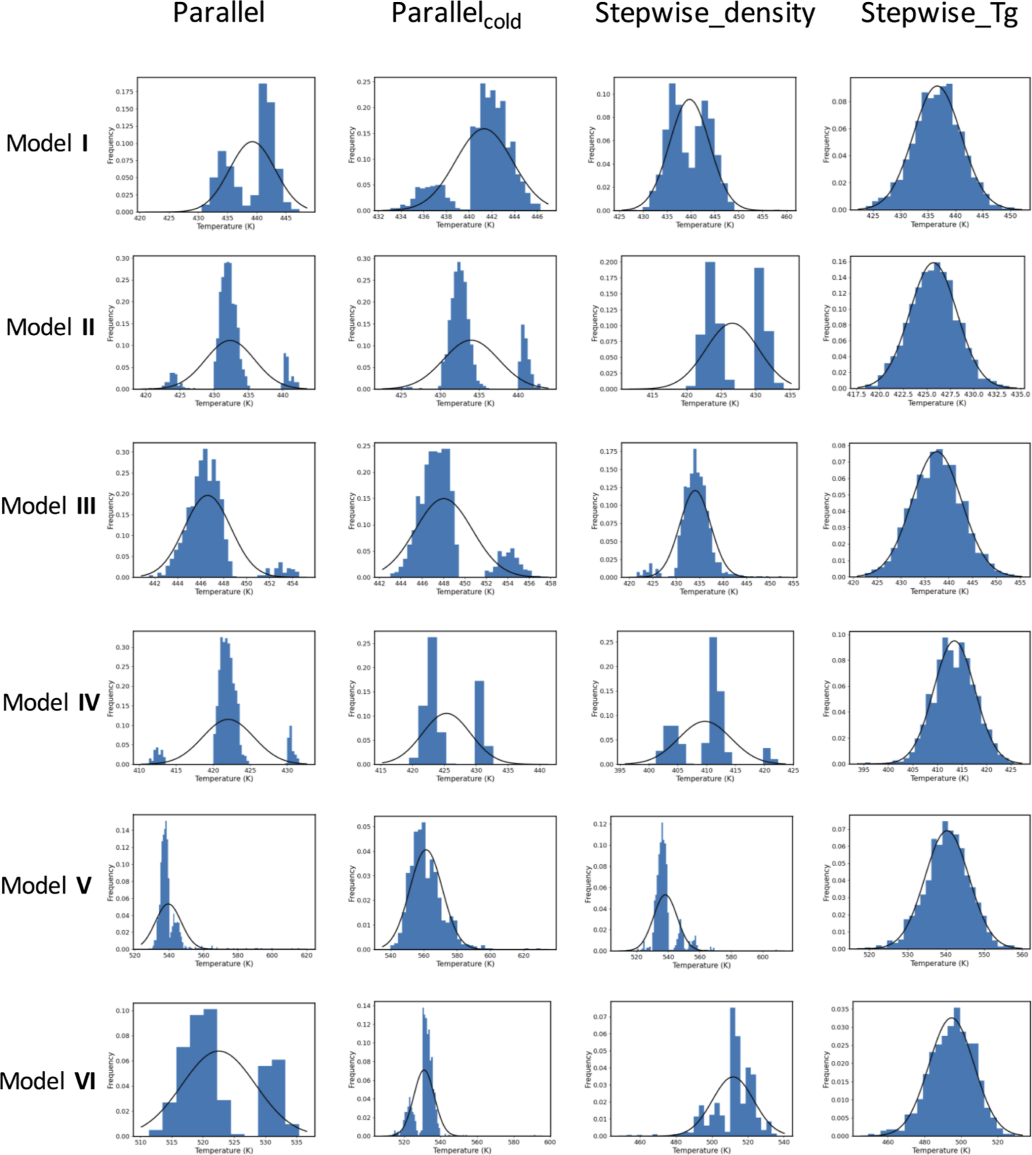
Distributions of *T*_g_ from the bootstrapped sampling of densities using the Patrone fitting method. The columns are the different protocols, as follows: (1) parallel; (2) parallel_cold; (3) stepwise_density_2ns; (4) stepwise_Tg_2ns. The systems are (a) DGEBA–44DDS (model **I**), (b) DGEBA–MDEA (model **II**), (c) DGEBF–44DDS (model **III**), (d) DGEBF–MDEA (model **IV**), (e) TGPAP–44DDS (model **V**), (f) TGPAP–MDEA (model **VI**). A fitted normal distribution is shown for each plot. For the majority of distributions, a multimodal distribution is observed.

In Figure 7 we show the distributions of the *T*_g_ predictions using the Patrone fitting method using bootstrapping analysis for the parallel, parallel_cold, stepwise_density_2ns and stepwise_Tg_2ns protocols. A normal distribution is especially clear for the distributions of *T*_g_ for the stepwise_Tg_2ns protocol; this is to be expected as we use bootstrapped sampling of the *T*_g_ computed for each stepwise replica and, as a result of the central-limit theorem, a Gaussian distribution is found. In the majority of the other cases that involve using bootstrap sampling of the densities, however, we find multimodal distributions. This multimodal distribution is also found for the *T*_g_ distributions computed using the fitting method of Lin et al. (Supporting Information Figure S6), indicating it is not an artifact of the fitting method of Patrone et al.

It is therefore instructive to look at the density distribution at each temperature point across the ensembles to determine why we find multimodal distributions for *T*_g_. In Supporting Information Table S7, we show the standard deviation, skew and excess kurtosis for the density distributions averaged across all temperatures for all resins and protocols. We find that the average excess kurtosis for the density distributions is negative. In Supporting Information Figure S11, we examine quantile–quantile (Q–Q) plots for a selection of systems, protocols and temperature points and their corresponding histograms. We observe that density distributions are often multimodal, with a wide distribution of values, which leads to the negative excess kurtosis. Interestingly, the average standard deviation across all systems is comparable, indicating that the stochastic uncertainty resulting from different initial conditions (i.e., atomic velocities) is invariant to the differing protocols for computing the density–temperature behavior. In Supporting Information Figure S12, we show that in the glassy state below *T*_g_, the variability in density computed by block averaging for a single simulation is much lower than across an ensemble. This indicates that using replicas with different initial conditions (i.e., velocities) leads to a sampling of metastable states for which transitions occur over a longer time scale than that of MD simulation. An example at 300 K is shown in Supporting Information Figure S13, where it is clear the variability across the ensemble is much higher than that of a single simulation.

**Figure 8:**
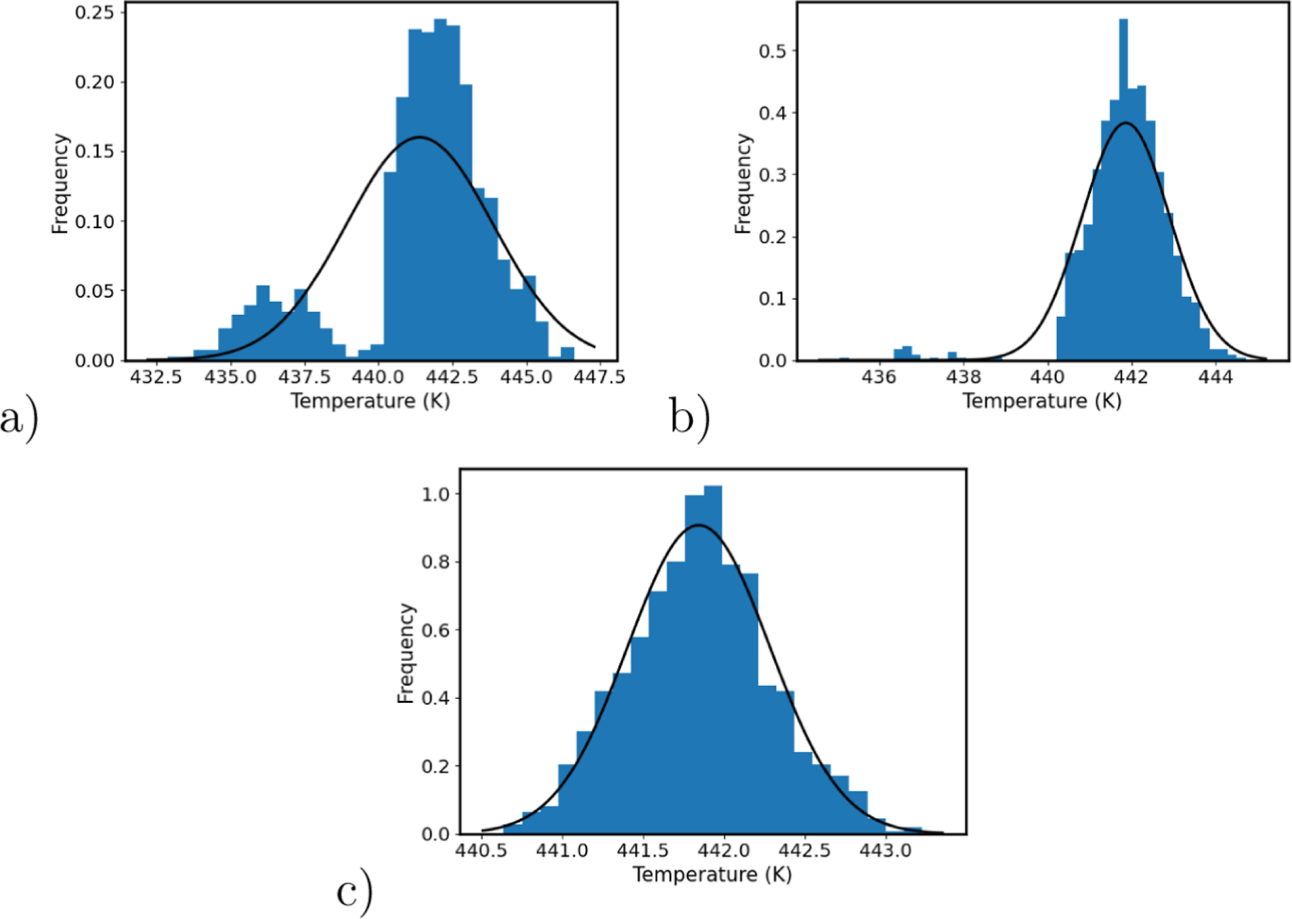
(a) *T*_g_ distributions from the stepwise_density_2ns protocol with Patrone fitting, using sampling from a normal distribution for each density point rather than bootstrap resampling. (a) Sampling from a normal distribution for each density point using with the standard deviation from the sample of 20 replicas. (b) Same as (a), but sampling from a normal distribution with a standard deviation scaled by 50%. (c) Same as (a), but with a normal distribution of 25%. With tighter density distributions in (c), we recover a normal distribution in *T*_g_.

To examine how the density distributions in the density–temperature curves can produce multimodal *T*_g_ distributions, we compared our results using bootstrap sampling with density values generated from random sampling of a normal distribution with different standard deviations (Figure 8).

Using the stepwise_density_2ns protocol for model **II** as example, using a standard deviation of 0.25 that of the actual density distribution leads to an approximately normal distribution of *T*_g_, while using the full standard deviation, we recover the multimodal *T*_g_ distribution seen in Figure 7 when using bootstrapped densities rather than a Gaussian model distribution. We conclude that, when the density distributions are wide enough, caused by the presence of multiple metastable states, the hyperbolic fitting of Patrone converges into distinct clusters. As a result, we observe multimodal *T*_g_ distributions. In Supporting Information Section 14, we show that higher *T*_g_ peaks have, in general, a higher ensemble-averaged density in the 80 K before the glass transition and a lower density in the 80 K after the glass transition.

To investigate the origins of the differences in densities across replicas, we have computed the correlation of density with other properties of the system (potential energy, van der Waals energy and coulomb energy) across the ensemble at each temperature. These correlation factors are given in Supporting Information Tables S8 and S9 and Figure S14. These correlation factors show that the density of the epoxy resin is directly correlated to the van der Waals interaction energy, indicating that favorable interactions result in more effective packing of the epoxy network. However, due to the complex nature of the cross-linked epoxy resin systems, we have found it is not possible to differentiate these global energetics into single dominant individual interaction contributions or conformations correlated to the density.

While not an objective of this study to analyze the microscopic transformations that occur to the epoxy during the glass transition, the large amount of conformational data produced by ensemble MD has allowed us to examine the changes in dynamical properties of the epoxy resin systems by computing the segmental relaxation times of bond vectors.^47^ This kind of study is always feasible because ensemble simulation typically generates up to an order of magnitude more data for analysis than one-off simulations do, again enhancing their statistical relevance. By presenting this analysis here so, we illustrate the postprocessing steps that can be realized after an ensemble MD campaign to provide some insight into the molecular origins of the glass transition. In Supporting Information Section 13, we find considerable slowdown in the dynamics of bond vectors involving the phenyl groups in the cross-linked resin at temperatures below the glass transition, while the bond-vectors involving the backbone atoms display predominantly Arrhenius behavior at all temperatures. This indicates that it is the structural relaxation of the aromatic rings that is characteristic of the glass transition.^48^

### 3.5 Density–Temperature Behavior

**Figure 9:**
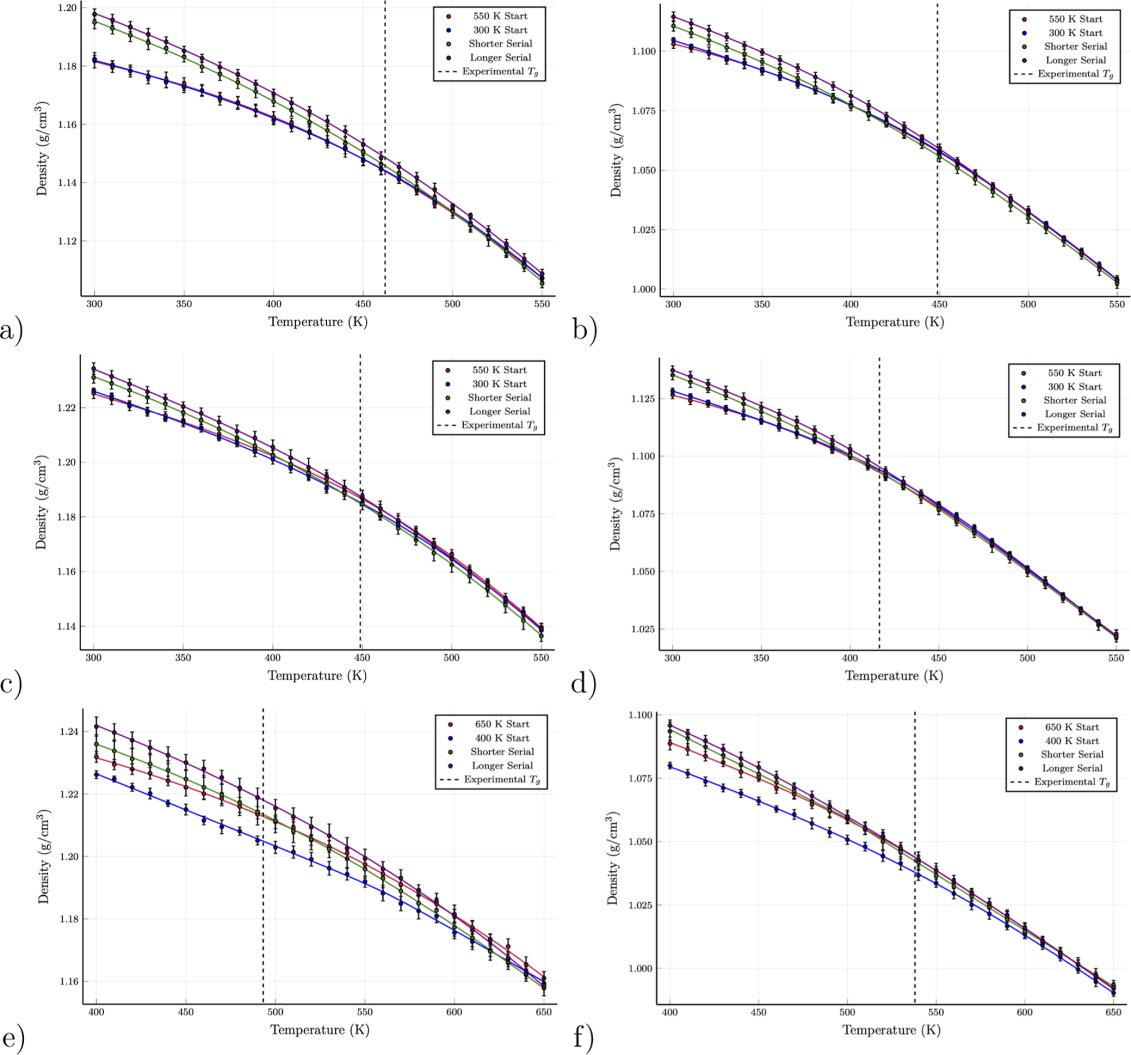
Density–temperature profiles computed by our various scenarios for (a) model **I** (DEGBA-44DDS), (b) model **II** (DGEBA–MDEA), (c) model **III** (DGEBF–44DDS), (d) model **IV** (DGEBF–MEA), (e) model **V** (TGPAP–44DDS), (f) model **VI** (TGPAP–MDEA).

The density–temperature curves for the six epoxy–resin systems are shown in Figure 9 for all protocols.

In every case, the densities using the parallel and parallel_cold protocols are lower than the stepwise protocols at lower temperatures. We can infer that this is due to the locking-in of high energy chain configurations when quenching to create the initial conditions for the parallel_cold and parallel protocols. In effect, the metastable state it achieves contains polymer configurations and free volume corresponding to elevated temperatures. The faster stepwise cooling rate (0.5 ns per 10 K) also shows lower densities at low temperatures than the slower cooling rate (2.0 ns per 10 K), although the densities are higher than with the parallel_cold and parallel protocols.

In Supporting Information Figure S17 we show a schematic diagram of common features of density–temperature curves of high and low *T*_g_ predictions; lower density in the glassy regime typically results in in higher *T*_g_ predictions. As the density is lower for parallel protocols compared to stepwise protocols in the glassy regime, we find higher *T*_g_ predictions for parallel protocols (Table 3).

The densities using parallel and parallel_cold protocols are identical for models **I–IV** but are significantly different for models **V** and **VI**, with parallel_cold protocols showing much lower densities than the parallel protocols at lower temperatures. Comparing the densities of models **I–IV** at 300 K to the experimental room temperature densities listed in Table 1, we find relatively good agreement using the stepwise_2ns protocols, which underestimate the density by between 1.5 and 3%, while the parallel protocols underestimate by 3–5%.

In summary, we have found that the stepwise scenarios create glassy systems with significantly higher density than the parallel scenarios. However, the difference in predicted *T*_g_ is relatively small (between 5 and 10 K), despite the difference in density–temperature curves with protocol. We can therefore conclude that the parallel protocols correctly sample changes in conformations (and hence detectable changes in density) around the glass transition point, even if the changes in density are greater for the stepwise scenarios. We attribute this to the hyperbolic fit of Patrone et al.,^42^ which uses the whole density–temperature curve, rather than just the intersection of high- and low-temperature density behavior. We also infer therefore that, since these protocols are detecting changes in the density–temperature behavior at *T*_g_, this is a general phenomenon and is not specific to the epoxy–resin systems considered in this study.

## 4 Conclusions

We have examined the aleatoric uncertainty associated with using classical MD to compute *T*_g_ for various computational scenarios. First, we conclude that ensembles are essential when using MD to compute *T*_g_, irrespective of the computational scenario used. Put in other words, we use the principles of statistical mechanics to calculate the glass transition temperature by ensemble averaging. We have shown that increasing the number of replicas within an ensemble reliably decreases the aleatoric uncertainty, while running individual replicas for longer times does not. The 95% confidence intervals for *T*_g_ of a single replica are approximately 60 and 80 K using the fitting methods of Patrone and Lin respectively, which reduces to below 16 and 40 K respectively for 20 replicas, depending on protocol used. For reliable and reproducible materials design, we recommend that at least 10 ensemble members be used with the Patrone fitting method to ensure the predicted *T*_g_ has a 95% confidence interval of less than 20 K. This should be the minimum requirement for any reported *T*_g_ using molecular dynamics (MD). This study adds to the growing body of evidence that ensembles are the most effective way to reduce aleatoric uncertainty; that running a large number of simulations and using ensemble averaged observables (such as the density) predicts *T*_g_ values with a much lower uncertainty than running a small number of simulations for longer.

Second, we conclude that highly parallel computational scenarios for computing glass transitions, where all temperatures are computed concurrently, produce *T*_g_ values that have small errors compared to experiment, and give predictions that are similar to traditional stepwise scenarios where each temperature is simulated sequentially. We therefore conclude that parallel scenarios can be used reliably to compute glass transition temperatures. We find no reduction in uncertainty by running MD simulations for longer than 6–7 ns in scenarios where all temperatures are run concurrently. Therefore, following this concurrent protocol, wall clock time can be reduced to only a few hours as opposed to days for traditional sequential approaches.

The success of the concurrent scenarios is due to the different initial velocity conditions for each member of the ensemble, which creates a distribution of densities significantly wider compared to one-off single MD simulations, those run for long periods of time (>10 ns). This is an example of the extreme sensitivity of classical molecular dynamics to initial conditions, resulting in the sampling of multiple relatively long-lived metastable states, averaging over which gives a better representation of the macroscopic sample. Using ensemble sampling, therefore, we can expect much better sampling of states where conformational changes associated with the glass transition occur. As a consequence, the density distributions at each temperature are often not normally distributed.

An explanation for the similarity in predicted values between the parallel and stepwise protocols is that, while the metastable states sampled in the stepwise protocols pass through an annealing process (i.e., they are cooled down from a higher temperature) due to the long-lived nature of the metastable states near and below the glass transition, the distribution of metastable states is similar to those sampled by directly simulating the system at the required temperature. Where the density distributions are wide, this can lead to multimodal distributions in the predicted *T*_g_, which would be missed with ensembles composed of small numbers of replicas, let alone one-off simulations.

This multimodal behavior is unlikely to be an artifact of insufficient sampling but rather a consequence of metastable states sampled from different initial conditions in phase space. In glassy systems with rugged energy landscapes, these states may deviate from a Boltzmann distribution, resulting in distinct peaks that reflect glass transitions between ensembles occupying different free-energy basins. Expanding the number of replicas could improve sampling and reveal whether a smooth, normal distribution of *T*_g_ values can be recovered. However, achieving this will likely require considerable computational resources due to the need for extensive state-space exploration. Despite the close agreement between the various scenarios, a difference in the density predictions in the glassy regime is observed, with significantly lower densities when all temperatures are simulated concurrently. However, the conformational changes that increase the density around the *T*_g_ are still detectable by the fitting method of Patrone et al. and hence the predictions are comparable across all scenarios. We conclude that if wall clock time is of paramount importance and accurate glassy densities are not required, the parallel scenario with the fitting method of Patrone et al. in an ensemble of at least ten replicas can lead to significant reductions in wall clock time: with 6 ns for total simulation time, the wall clock time is less than 12% of the stepwise_2ns scenario, which for our resin models equates to several hours instead of 2 days. These timings could be further reduced if graphics processing unit (GPU) accelerated molecular dynamics is used. This result is highly important when turnaround time is key, for example for high throughput screening of candidate polymers. Of course, due to the independent nature of the replicas in ensemble MD approaches, there is no requirement for all replicas to be run concurrently if sufficient resources for so doing are not available.

The focus of this paper was to examine the aleatoric uncertainty when using MD simulations to predict glass transition temperatures, and to suggest methods to reduce this uncertainty. There are numerous parameters associated with the simulations whose effects could be assessed. In this paper, we have restricted ourselves to a few common variables (such as number of MD simulations in an ensemble, data collection times and the method to extract *T*_g_ from the density–temperature curves). Future work could examine the significance of other variables, such as the size of the simulation box, methods of initialization, chemical connectivity, force field parameters, starting temperature and so on.

We have previously shown the role of different parametric inputs in the computation of the Young’s modulus and Poisson ratio of related epoxy resins by deriving an active subspace, which reduced the dimensionality from well in excess of one hundred to one (itself a linear combination of those parameters whose uncertainties contribute significantly to the sensitivity of the quantity of interest).^33^

This required both the invention and implementation of new and scalable UQ methods (based on active subspaces that invoke machine learning and Gaussian processes) to determine the sensitivity of the input parameters. In this computationally expensive study over 100 force field parameters were assessed, which we showed may be ranked in terms of their importance to the uncertainties they confer on the materials properties. Applying this approach would amount to a substantial body of work which would build upon the present study that is focused on aleatoric uncertainty, extending it to embrace parametric uncertainty. In addition, these techniques will be able to quantify the role of the force field terms and that in turn will reveal which energetic parameters provide the most crucial input in determining *T*_g_.

## 5 Computational Methods

### 5.1 Model Building

The initial structure of the six cross-linked epoxy resins were built using the MedeA Amorphous Materials Thermoset Builder software from Materials Design.^49^ The MedeA thermoset builder works by an iterative process which forms bonds between the epoxy and amine molecules, starting from a random arrangement of precursor molecules. At every step of the builder, it searches nearby molecules in an increasing radius in order to form bonds between them. This is followed by energy minimization steps at constant temperature and volume (*NVT*). This builder is similar to other simulated atomistic cross-linkers described in the literature.^50−53^ The epoxies that are being investigated are DGEBA, DBEGF, and TGPAP, which are cross-linked to the amines 44DDS and MDEA for a total of six systems (see Figure 1). The epoxies and amines were mixed at a stoichiometry of 100% (i.e., a ratio of 2:1 for DGEBA/DGEBF to 44DDS/MDEAmolecules and a ratio 4:3 for TGPAP to 44DDS/MDEA) in order to allow the possibility of a fully cured system. The cross-linking ratio has a considerable impact on the density as well as the temperature of the glass transition. In order to achieve consistency between the different system, the cross-linking process was stopped when exactly 95% of the epoxy groups have formed bonds with the amines.

After cross-linking, the potential energy of systems was minimized by adjusting the atom coordinates using steepest descent with quadratic line search. The number of atoms for each model is between 23,000 and 32,000 atoms (see Supporting Information Section 2 for more details of each model and Supporting Information Figure S1 for a visualization of the simulation box). This is followed by running MD under a constant pressure of 1 atm for 13 ns at a fixed temperatures of 300 K for the parallel_cold protocol and 550 K for parallel protocol for the molecules with two epoxy groups (models **I**–**IV**), and 400 K (parallel_cold) and 650 K (parallel) for TGPAP, which has three epoxy groups (models **V** and VI) respectively. This creates the initial structures for use in the scenarios detailed in Section 2.1. In Section 4 of the Supporting Information, we have shown that the densities and potential energies of all models have reached constant values with no observable drift, before the end of the 13 ns elevated temperature simulation. In addition, for all simulations, we have computed the mean squared difference in the radial distribution functions (RDFs) for all outputted timesteps with the final time step in the data collection time frame. To ensure the structure is stable, a linear regression was fitted to these differences and the slope of any detected trend was checked such that it was less than the standard deviation of the mean squared differences in RDFs.

### 5.2 Molecular Dynamics

The parallel protocols are as follows: starting at final snapshot of the MD simulation at the highest temperature, the system is cloned into identical replicas with the velocities initialized with different random seeds. For each system, the temperature is then changed to a different temperature under constant volume through *NVT*. Finally, the systems are simulated at their new temperatures under constant temperature and pressure (*NPT*) using the Nosé–Hoover Thermo/Barostat. The time step used was 1.0 fs. In this study, for the parallel scenarios, the *NVT* step used to change the temperature was performed for 0.2 ns and the *NPT* simulation was subsequently run for 13 ns. For the stepwise scenarios, we investigated the range of temperature from 300 to 550 K for the systems composed of molecules with two epoxy groups and a range of 400 to 650 K for the molecules with three epoxy groups. In both cases the temperature points were separated by increments of 10 K and a total of 26 temperature points were run.

The MD simulations were performed using the LAMMPS^54^ software package with the all-atom class II force field PCFF+.^55−57^ PCFF+ has been shown to be effective in predicting glass transition temperatures. Studying the biopolymers Polyhydroxyalkanoates^58^ examined three force fields: the CHARMM General Force Field (CGenFF), the Generalized Amber Force Field (GAFF) and the Polymer Consistent Force Field (PCFF+), validating them against experimental results. The PCFF + significantly outperforms the other two force fields and predicts a value of the *T*_g_ with an error of 16.1% as compared to 44.5% and 48.8% for the CHARMM and Amber force fields, respectively.^58^ The short-range cutoff for nonbonded interactions is 9.5 Å. The long-range Coulombic interactions were computed using the particle–particle–particle mesh method. Atomic positions were outputted every 0.1 ns. Densities were outputted every 1 ps.

All simulations are performed with periodic boundary conditions; the cross-linked molecules pass through the periodic boundaries. The post analysis and graphical output is produced using the Julia programming language^59^ and python. The python codes used routines from EasyVVUQ^20^ to perform the bootstrapping, part of the VECMA Toolkit.^27,60^

## 6 Experimental Methods

Glass transition temperatures were determined by the authors using dynamic mechanical analysis (DMA). DMA measures the mechanical response of the sample to an applied oscillatory force at set frequency as a function of temperature. The glass transition temperature is determined through a peak in the tan δ curve, which represents the ratio of the loss modulus to the storage modulus measured during the DMA experiment. We used a TA Q800 instrument on cured resin specimens that were 2 mm in thickness, 8 mm wide and approximately 40 mm long. A single cantilever fixture was used to mount the specimen. Measurements were carried out from 25 to 250 °C at a ramp rate of 5 °C/min, at a frequency of 1 Hz and using an amplitude of 30 mm. The experiment was repeated between 4 and 10 times to give the standard deviations listed in Table 1.

## Data Availability

LAMMPS input files, structures, parameters and output files, and analysis scripts are openly available at 10.5281/zenodo.11073749.

## Supporting Information Available

Additional discussions of experimental uncertainty in determining *T*_g_, numerical details of *T*_g_ predictions for each model, including as a function of ensemble size and length of simulation/data collection, analysis of density distributions at different temperature points, segmental relaxation times, mean-squared displacements (PDF)

## References

[ref1] Sharp N.; Li C.; Strachan A.; Adams D.; Pipes R. B. Effects of water on epoxy cure kinetics and glass transition temperature utilizing molecular dynamics simulations. J. Polym. Sci., Part B: Polym. Phys. 2017, 55, 1150–1159. 10.1002/polb.24357.

[ref2] Yadav A.; Kumar A.; Singh P. K.; Sharma K. Glass transition temperature of functionalized graphene epoxy composites using molecular dynamics simulation. Integr. Ferroelectr. 2018, 186, 106–114. 10.1080/10584587.2017.1370331.

[ref3] Makarov G.; Borodina O.; Makarova T.; Ignatova A.; Olivenko N.; Bartashevich E.; Sapozhnikov S. Molecular Dynamics Study of Cured ED-20 Epoxy Resin for Predicting the Glass Transition Temperature and Relationship with Structure Features. J. Phys. Chem. A 2023, 127, 3894–3905. 10.1021/acs.jpca.3c00425. 37083410

[ref4] Ji W.; Zhang L. Diamond nanothread reinforced polymer composites: ultra-high glass transition temperature and low density. Compos. Sci. Technol. 2019, 183, 107789 10.1016/j.compscitech.2019.107789.

[ref5] Chowdhury S. C.; Elder R. M.; Sirk T. W.; Gillespie J. W. Epoxy resin thermo-mechanics and failure modes: Effects of cure and cross-linker length. Composites, Part B 2020, 186, 107814 10.1016/j.compositesb.2020.107814.

[ref6] Chowdhury S. C.; Prosser R.; Sirk T. W.; Elder R. M.; Gillespie J. W. Glass fiber-epoxy interactions in the presence of silane: A molecular dynamics study. Appl. Surf. Sci. 2021, 542, 148738 10.1016/j.apsusc.2020.148738.

[ref7] Martí D.; Pétuya R.; Bosoni E.; Dublanchet A.-C.; Mohr S.; Léonforte F. Predicting the Glass Transition Temperature of Biopolymers via High-Throughput Molecular Dynamics Simulations and Machine Learning. ACS Appl. Polym. Mater. 2024, 6 ( (8), ), 4449–4461.

[ref8] Srikanth A.; Abrams C. F. Effect of molecular packing and hydrogen bonding on the properties of epoxy-amido amine systems. Comput. Mater. Sci. 2019, 169, 109082 10.1016/j.commatsci.2019.109082.

[ref9] Yang J. H.; Srikanth A.; Jang C.; Abrams C. F. Relationships between molecular structure and thermomechanical properties of bio-based thermosetting polymers. J. Polym. Sci., Part B: Polym. Phys. 2017, 55, 285–292. 10.1002/polb.24270.

[ref10] Bejagam K. K.; Iverson C. N.; Marrone B. L.; Pilania G. Molecular dynamics simulations for glass transition temperature predictions of polyhydroxyalkanoate biopolymers. Phys. Chem. Chem. Phys. 2020, 22, 17880–17889. 10.1039/D0CP03163A. 32776023

[ref11] Klajmon M.; Aulich V.; Ludík J.; Červinka C. Glass Transition and Structure of Organic Polymers from All-Atom Molecular Simulations. Ind. Eng. Chem. Res. 2023, 62, 21437–21448. 10.1021/acs.iecr.3c03038.

[ref12] Bezik C. T.; Redline E. M.; Foster J. C.; Frischknecht A. L. Simulations of Glass Transition and Mechanical Behavior of Off-Stoichiometric Crosslinked Polymers. Macromolecules 2023, 56, 5268–5277. 10.1021/acs.macromol.3c00924.

[ref13] Khare K. S.; Phelan F. R. Integration of Atomistic Simulation with Experiment Using Time- Temperature Superposition for a Cross-Linked Epoxy Network. Macromol. Theory Simul. 2020, 29, 1900032 10.1002/mats.201900032.

[ref14] Sirk T. W.; Karim M.; Lenhart J. L.; Andzelm J. W.; Khare R. Bi-modal polymer networks: Viscoelasticity and mechanics from molecular dynamics simulation. Polymer 2016, 90, 178–186. 10.1016/j.polymer.2016.03.024.

[ref15] Huang M.; Abrams C. Effects of reactivity ratios on network topology and thermomechanical properties in vinyl ester/styrene thermosets: molecular dynamics simulations. Macromol. Mater. Eng. 2019, 28, 1900030 10.1002/mats.201900030.

[ref16] Jian W.; Wang X.; Lu H.; Lau D. Molecular dynamics simulations of thermodynamics and shape memory effect in CNT-epoxy nanocomposites. Compos. Sci. Technol. 2021, 211, 108849 10.1016/j.compscitech.2021.108849.

[ref17] Afzal M. A. F.; Browning A. R.; Goldberg A.; Halls M. D.; Gavartin J. L.; Morisato T.; Hughes T. F.; Giesen D. J.; Goose J. E. High-throughput molecular dynamics simulations and validation of thermophysical properties of polymers for various applications. ACS Appl. Polym. Mater. 2021, 3, 620–630. 10.1021/acsapm.0c00524.

[ref18] Wan S.; Bhati A. P.; Wade A. D.; Coveney P. V. Ensemble-Based Approaches Ensure Reliability and Reproducibility. J. Chem. Inf. Model. 2023, 63, 6959–6963. 10.1021/acs.jcim.3c01654. 37965695 PMC10685440

[ref19] Wan S.; Sinclair R. C.; Coveney P. V. Uncertainty quantification in classical molecular dynamics. Philos. Trans. R. Soc., A 2021, 379, 20200082 10.1098/rsta.2020.0082. PMC8059622 33775140

[ref20] Wright D. W.; Richardson R. A.; Edeling W.; Lakhlili J.; Sinclair R. C.; Jancauskas V.; Suleimenova D.; Bosak B.; Kulczewski M.; Piontek T.; et al. Building Confidence in Simulation: Applications of EasyVVUQ. Adv. Theory Simul. 2020, 3, 1900246 10.1002/adts.201900246.

[ref21] Coveney P. V.; Wan S. On the calculation of equilibrium thermodynamic properties from molecular dynamics. Phys. Chem. Chem. Phys. 2016, 18, 30236–30240. 10.1039/C6CP02349E. 27165501

[ref22] Wan S.; Bhati A. P.; Zasada S. J.; Coveney P. V. Rapid, accurate, precise and reproducible ligand–protein binding free energy prediction. Interface Focus 2020, 10, 20200007 10.1098/rsfs.2020.0007. 33178418 PMC7653346

[ref23] Bhati A. P.; Wan S.; Wright D. W.; Coveney P. V. Rapid, accurate, precise, and reliable relative free energy prediction using ensemble based thermodynamic integration. J. Chem. Theory Comput. 2017, 13, 210–222. 10.1021/acs.jctc.6b00979. 27997169

[ref24] Palmer T. The ECMWF ensemble prediction system: Looking back (more than) 25 years and projecting forward 25 years. Q. J. R. Meteorol. Soc. 2019, 145, 12–24. 10.1002/qj.3383.

[ref25] Coveney P. V.; Groen D.; Hoekstra A. G. Reliability and reproducibility in computational science: Implementing validation, verification and uncertainty quantification in silico. Philos. Trans. R. Soc., A 2021, 379, 20200409 10.1098/rsta.2020.0409. 33775138

[ref26] Richardson R. A.; Wright D. W.; Edeling W.; Jancauskas V.; Lakhlili J.; Coveney P. V. EasyVVUQ: a library for verification, validation and uncertainty quantification in high performance computing. J. Open Res. Software 2020, 8, 11 10.5334/jors.303.

[ref27] Groen D.; Richardson R. A.; Wright D. W.; Jancauskas V.; Sinclair R.; Karlshoefer P.; Vassaux M.; Arabnejad H.; Piontek T.; Kopta P. Introducing VECMAtk: verification, validation and uncertainty quantification for multiscale and HPC simulations. Computational Science-ICCS 2019: 19th International Conference, Faro, Portugal, June 12–14, 2019, Proceedings, Part IV 19, 2019; pp 479–492.

[ref28] Patrone P. N.; Dienstfrey A. Reviews in Computational Chemistry; John Wiley & Sons: Hoboken, NJ, USA, 2018; Vol. 31, pp 115–169.

[ref29] Vassaux M.; Wan S.; Edeling W.; Coveney P. V. Ensembles Are Required to Handle Aleatoric and Parametric Uncertainty in Molecular Dynamics Simulation. J. Chem. Theory Comput. 2021, 17, 5187–5197. 10.1021/acs.jctc.1c00526. 34280310 PMC8389531

[ref30] Edeling W.; Arabnejad H.; Sinclair R.; Suleimenova D.; Gopalakrishnan K.; Bosak B.; Groen D.; Mahmood I.; Crommelin D.; Coveney P. V. The impact of uncertainty on predictions of the CovidSim epidemiological code. Nat. Comput. Sci. 2021, 1, 128–135. 10.1038/s43588-021-00028-9. 38217226

[ref31] Minisini B.; Soldera A. Volumetric and Energetic Properties of Polystyrene and Polyethylene Oxide Affected by Thermal Cycling. Macromol. Theory Simul. 2023, 32, 2300008 10.1002/mats.202300008.

[ref32] Hutchinson J. Determination of the glass transition temperature: Methods correlation and structural heterogeneity. J. Therm. Anal. Calorim. 2009, 98, 579–589. 10.1007/s10973-009-0268-0.

[ref33] Edeling W.; Vassaux M.; Yang Y.; Wan S.; Guillas S.; Coveney P. V. Global ranking of the sensitivity of interaction potential contributions within classical molecular dynamics force fields. npj Comput. Mater. 2024, 10, 87 10.1038/s41524-024-01272-z.

[ref34] Kumar S.; Krishnan S.; Samal S. K.; Mohanty S.; Nayak S. K. Toughening of Petroleum Based (DGEBA) Epoxy Resins with Various Renewable Resources Based Flexible Chains for High Performance Applications: A Review. Ind. Eng. Chem. Res. 2018, 57, 2711–2726. 10.1021/acs.iecr.7b04495.

[ref35] Kwon S. H.; Kang H.; Kim B.-J.; Lee H. I.; Lee J. M.; Kim J.; Lee S. G. Addressing diffusion behavior and impact in an epoxy–amine cure system using molecular dynamics simulations. Sci. Rep. 2023, 13, 138 10.1038/s41598-022-26835-2. 36599868 PMC9813372

[ref36] Banerjee A.; Hsu H.-P.; Kremer K.; Kukharenko O. Data-driven identification and analysis of the glass transition in polymer melts. ACS Macro Lett. 2023, 12, 679–684. 10.1021/acsmacrolett.2c00749. 37167550 PMC10286554

[ref37] Banerjee A.; Iscen A.; Kremer K.; Kukharenko O. Determining glass transition in all-atom acrylic polymeric melt simulations using machine learning. J. Chem. Phys. 2023, 159, 074108 10.1063/5.0151156. 37602800

[ref38] Iwaoka N.; Takano H. Conformational fluctuations of polymers in a melt associated with glass transition. J. Phys. Soc. Jpn. 2017, 86, 035002 10.7566/JPSJ.86.035002.

[ref39] Underwood T.; Purton J.; Manning J.; Brukhno A.; Stratford K.; Düren T.; Wilding N.; Parker S. dlmontepython: A Python library for automation and analysis of Monte Carlo molecular simulations. 2021, arXiv preprint: arXiv.2104.03822.

[ref40] pymbar. https://github.com/choderalab/pymbar, (accessed March 24, 2024).

[ref41] Chodera J. D. A simple method for automated equilibration detection in molecular simulations. J. Chem. Theory Comput. 2016, 12, 1799–1805. 10.1021/acs.jctc.5b00784. 26771390 PMC4945107

[ref42] Patrone P. N.; Dienstfrey A.; Browning A. R.; Tucker S.; Christensen S. Uncertainty quantification in molecular dynamics studies of the glass transition temperature. Polymer 2016, 87, 246–259. 10.1016/j.polymer.2016.01.074.

[ref43] Lin K.-H.; Paterson L.; May F.; Andrienko D. Glass transition temperature prediction of disordered molecular solids. npj Comput. Mater. 2021, 7, 179 10.1038/s41524-021-00647-w.

[ref44] Carmona Esteva F. J.; Zhang Y.; Colón Y. J.; Maginn E. J. Molecular dynamics simulation of the influence of external electric fields on the glass transition temperature of the ionic liquid 1-ethyl-3-methylimidazolium bis (trifluoromethylsulfonyl) imide. J. Phys. Chem. B 2023, 127, 4623–4632. 10.1021/acs.jpcb.3c00936. 37192465

[ref45] Klajmon M.; Červinka C. Does Explicit Polarizability Improve Molecular Dynamics Predictions of Glass Transition Temperatures of Ionic Liquids?. J. Phys. Chem. B 2022, 126, 2005–2013. 10.1021/acs.jpcb.1c10809. 35195429

[ref46] Gavrielides A.; Duguet T.; Aufray M.; Lacaze-Dufaure C. Model of the DGEBA-EDA Epoxy Polymer: Experiments and Simulation Using Classical Molecular Dynamics. Int. J. Polym. Sci. 2019, 2019, 1–9. 10.1155/2019/9604714.

[ref47] Siachouli P.; Karadima K. S.; Mavrantzas V. G.; Pandis S. N. The effect of functional groups on the glass transition temperature of atmospheric organic compounds: a molecular dynamics study. Soft Matter 2024, 20, 4783–4794. 10.1039/D4SM00405A. 38847330

[ref48] Vuković F.; Swan S. R.; Reyes L. Q.; Varley R. J.; Walsh T. R. Beyond the ring flip: A molecular signature of the glass–rubber transition in tetrafunctional epoxy resins. Polymer 2020, 206, 122893 10.1016/j.polymer.2020.122893.

[ref49] Materials Design. MedeA version 3.0; MedeA is a Registered Trademark of Materials Design Inc.: San Diego, USA. https://www.materialsdesign.com/medea-software.

[ref50] Hall S. A.; Howlin B. J.; Hamerton I.; Baidak A.; Billaud C.; Ward S. Solving the Problem of Building Models of Crosslinked Polymers: An Example Focussing on Validation of the Properties of Crosslinked Epoxy Resins. PLoS One 2012, 7, e42928 10.1371/journal.pone.0042928. 22916182 PMC3423435

[ref51] Paajanen A.; Vaari J.; Verho T. Crystallization of cross-linked polyethylene by molecular dynamics simulation. Polymer 2019, 171, 80–86. 10.1016/j.polymer.2019.03.040.

[ref52] Demir B.; Walsh T. R. A robust and reproducible procedure for cross-linking thermoset polymers using molecular simulation. Soft Matter 2016, 12, 2453–2464. 10.1039/C5SM02788H. 26822527

[ref53] Xie Q.; Liang S.; Liu B.; Fu K.; Zhan Z.; Lu L.; Yang X.; Lü F.; Huang Z. Structure, microparameters and properties of crosslinked DGEBA/MTHPA: A molecular dynamics simulation. AIP Adv. 2018, 8, 075332 10.1063/1.5041283.

[ref54] Thompson A. P.; Aktulga H. M.; Berger R.; Bolintineanu D. S.; Brown W. M.; Crozier P. S.; in’t Veld P. J.; Kohlmeyer A.; Moore S. G.; Nguyen T. D.; Shan R.; Stevens M. J.; Tranchida J.; Trott C.; Plimpton S. J. LAMMPS - a flexible simulation tool for particle-based materials modeling at the atomic, meso, and continuum scales. Comput. Phys. Commun. 2022, 271, 108171 10.1016/j.cpc.2021.108171.

[ref55] Sun H.; Mumby S. J.; Maple J. R.; Hagler A. T. An ab-initio CFF93 All-Atom Force Field for Polycarbonates. J. Am. Chem. Soc. 1994, 116, 2978–2987. 10.1021/ja00086a030.

[ref56] Maple J. R.; Hwang M.-J.; Stockfisch T. P.; Dinur U.; Waldman M.; Ewig C. S.; Hagler A. T. Derivation of class II force fields. I. Methodology and quantum force field for the alkyl functional group and alkane molecules. J. Comput. Chem. 1994, 15, 162–182. 10.1002/jcc.540150207.

[ref57] Hwang M. J.; Stockfisch T. P.; Hagler A. T. Derivation of Class II Force Fields. 2. Derivation and Characterization of a Class II Force Field, CFF93, for the Alkyl Functional Group and Alkane Molecules. J. Am. Chem. Soc. 1994, 116, 2515–2525. 10.1021/ja00085a036.

[ref58] Bejagam K. K.; Iverson C. N.; Marrone B. L.; Pilania G. Molecular dynamics simulations for glass transition temperature predictions of polyhydroxyalkanoate biopolymers. Phys. Chem. Chem. Phys. 2020, 22, 17880–17889. 10.1039/D0CP03163A. 32776023

[ref59] Bezanson J.; Edelman A.; Karpinski S.; Shah V. B. Julia: A Fresh Approach to Numerical Computing. SIAM Rev. 2017, 59, 65–98. 10.1137/141000671.

[ref60] Groen D.; Arabnejad H.; Jancauskas V.; Edeling W.; Jansson F.; Richardson R. A.; Lakhlili J.; Veen L.; Bosak B.; Kopta P.; et al. VECMAtk: a scalable verification, validation and uncertainty quantification toolkit for scientific simulations. Philos. Trans. R. Soc., A 2021, 379, 20200221 10.1098/rsta.2020.0221. PMC8059654 33775151

