# Supplementary material for: Rapid, Accurate and Reproducible Prediction of the Glass Transition Temperature Using Ensemble-Based Molecular Dynamics Simulation
Source: J Chem Theory Comput. 2025 Jan 29;21(3):1405–21. doi: 10.1021/acs.jctc.4c01364 (PMC11823416; doi:10.1021/acs.jctc.4c01364)
Supplement: Supplementary file 1 — ct4c01364_si_001.pdf [file ct4c01364_si_001.pdf]

# Supporting Information: Rapid, accurate and reproducible prediction of the glass transition temperature using ensemble-based molecular dynamics simulation

James L. Suter 0000-0002-0149-7974,[†] Werner A. Müller,[†] Maxime Vassaux,[‡] Alexandros Anastasiou,[¶] Martin Simmons,[¶] David Tilbrook,[¶] and Peter V. Coveney 0000-0002-8787-7256[*,†,§,‖]

†*Centre for Computational Science - University College London, 20 Gordon Street, London, WC1H 0AJ, United Kingdom*

‡*Institute of Physics Rennes, CNRS, IPR- UMR 6251, F-35000*

¶*Hexcel Composites, Ickleton Road, Duxford, Cambridge, Cambridgeshire, CB22 4QD, United Kingdom*

§*Advanced Research Computing Centre, University College London, London, WC1E 6BT, United Kingdom*

‖*Computational Science Laboratory, Institute for Informatics, Faculty of Science, University of Amsterdam, 1098XH, The Netherlands*

E-mail: p.v.coveney@ucl.ac.uk

In this Supporting Information (SI), we discuss further the uncertainty associated with experimental determination of $T_g$. We also present additional information about the systems simulated in this study and report in more detail on our predictions of $T_g$, which are sum-

marised in the main paper.

## 1 DISCUSSION OF EXPERIMENTAL $T_g$ OF DGEBA-44DDS

To illustrate the uncertainty associated with experimentally derived values for $T_g$ we briefly discuss the wide range of reported degrees of cross-linking and $T_g$ of the DGEBA-44DDS epoxy resin. The degree of cross-linking has a dramatic effect: White *et al.* showed a shift from 179$^o$C to 190$^o$C when increasing from 90% to 100% maximum achievable cure state of DGEBA-44DDS[1].

The degree of cross-linking in the study of White *et al.* was set by the controlling the relative weights of epoxy and amine in the mixture such that stoichiometric quantities gives the maximum achievable cure state. However, whether this results in all epoxy groups reacting is still currently unknown, and hence the actual degree of cross-linking in the sample studied is not known. Alessi *et al.*[2] estimated the degree of cross-linking from examining changes in the solid-state NMR spectra of DGEBA-44DDS to determine the percentage of un-reacted epoxy rings. The percentage of open epoxy rings for the as-synthesized samples increased with the curing time, reaching 62% after curing at 180$^o$ C for 120 minutes. However, it is common for the curing time to be longer and at higher temperatures. This will increase the amount of cross-linking, as shown by increased $T_g$. Min *et al.* found that $T_g$ reached a maximum at 207$^o$ C - 217$^o$ C, depending on the experimental method used, with a cure time of 6 hours. Marks *et al.* used an approach that relates changes in the thermoset heat capacity and $T_g$ to the degree of cross-linking and found that curing at TGEBA-44DDS at 220$^o$C for 1 hour leads to full conversion[3]. In Li *et al.* it was reported that FTIR data for the related DGEBA-33DDS system indicates that 92% conversion is reached for a 1:1 stiochometric system[4]. This illustrates that model structure uncertainty can make validation with experiment problematical, as the degree of crosslinking is not only hard to determine, but also very sensitive to the procedure used to cure the system.

When comparing to experiment for validation, we also have to consider experimental sce-

nario uncertainty due to the choice of experimental procedure and protocol. There are three common methods used to determine the glass transition temperature: Differential Scanning Calorimetry (DSC), where the $T_g$ is determined by a change in heat capacity as a function of temperature; Dynamic Mechanical Analysis (DMA), where changes in the mechanical properties (loss modulus or tangent delta) as a function of temperature or frequency are used to determine $T_g$ and Thermomechanical Analysis (TMA), where $T_g$ is often determined by the onset of thermal expansion as a function of temperature change. These methods can lead to significantly different reported $T_g$. For example, White *et al.* reported a $T_g$ of 207$^o$C when using DSC method for the fully cured DGEBA-44DDS sample, while the equivalent sample produced a $T_g$ of 190$^o$C using Dynamic Mechanical Analysis with an oscillation frequency of 1 Hz[1]. The oscillation frequency in DMA analysis can also alter the reported $T_g$; this is the frequency of the sinusoidal oscillatory deformation applied to the sample, with lower frequencies leading to a lower $T_g$. For example, the reported $T_g$ of PET films varied from 119.2$^o$C to 109.4$^o$C as the frequency was changed from 10Hz to 0.3Hz[5].

## 2 EPOXY MODEL SYSTEMS

In Table S1, we list the number of atoms, initial and final supercell lattice dimensions after 13ns for the parallel protocols, and at 2ns for stepwise_2ns protocols at their lowest simulated temperature (300K for models **I-IV** and 400K for models **V-VI**). The initial lattice dimensions are after 13ns simulation at the highest temperature (550K for models **I-IV** and 650K for models **V-VI**). The lattice dimensions in Table S1 are averaged across the ensemble. Note that all three lattice dimensions ($x$, $y$, $z$) in the simulation supercell are constrained to be the same during the MD simulation, that is, they dilate/contract together in response to the external pressure (set in the Nosé-Hoover barostat to be 1 atmosphere). The standard deviations reported in Table S1 correspond to the variation across the ensemble of 20 replicas.

Table S1: Details of the epoxy resin systems studied in this paper.

| Resin | Number of atoms | Initial lattice dimensions (Å) | Parallel protocol final lattice dimensions at 300/400K (Å) | Stepwise_2ns protocol final lattice dimensions at 300/400K (Å) |
|---|---|---|---|---|
| **I** | 25480 | 65.6 | 63.9 ± 0.06 | 63.6 ± 0.06 |
| **II** | 30280 | 68.8 | 66.8 ± 0.06 | 66.6 ± 0.06 |
| **III** | 23080 | 63.5 | 61.9 ± 0.03 | 61.7 ± 0.04 |
| **IV** | 27880 | 67.2 | 65.1 ± 0.06 | 64.8 ± 0.06 |
| **V** | 24420 | 64.3 | 63.0 ± 0.04 | 62.8 ± 0.06 |
| **VI** | 31620 | 69.8 | 67.8 ± 0.07 | 67.6 ± 0.08 |

## 3 VISUALISATION OF THE EPOXY-RESIN SYSTEM

A snapshot from one of the replicas of the model **I** system simulated at 300K is shown in Figure S1.

## 4 ESTIMATION OF THE GLASS TRANSITION TEMPERATURE USING AUTOMATED METHODS OF DETERMINING STEADY-STATE

We have compared our predicted $T_g$ values using density collection during set times (12-13ns for the parallel protocols, 1-2ns and 0.1-0.5ns for stepwise_2ns and stepwise_0.5ns protocols respectively) with density collection after a steady-state (on the timescale of our MD simulations) has been determined to occur. We have used two programmes for automatically determining when a steady state has been reached (dlmontepython[7] and pymbar[8,9]). More details on their heuristics for determining when a steady-state has been reached can be found in the relevant publications. We have used default parameters, as described in the publications and in the code.

In Table S2, we list the predicted $T_g$ values using density values averaged from the steady-state detection time computed by each programme to the end of the simulation. Comparing to predicted $T_g$ values using set density collection times (Table 3 in the main paper), and those with differing start and end points of data collection (Figures S9 and S10), we find all values are within one standard deviation of each other.

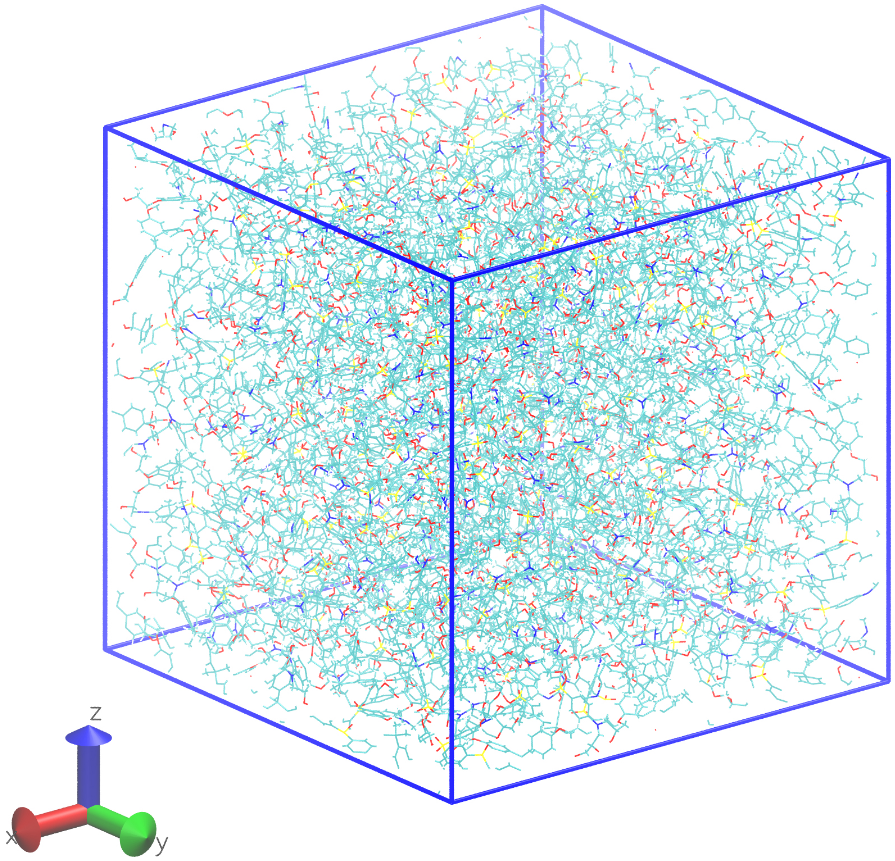


Figure S1: Snapshot of one of the replicas of model **I** at 300K visulated using VMD[6], illustrating the cross-linked molecule fills the simulation box and crosses over the periodic boundaries. The periodic boundaries are shown as blue lines. The atom colours are as follows: green = carbon atoms, blue = nitrogen, red = oxygen, sulphur = yellow, white = hydrogen.

To illustrate the evolution of the system density with time, and the determination of steady-state, in Figure S2 we have plotted the density with respected to time for a selected replica of Model **I** using the parallel protocol at 300K and 550K, and indicated the steady-state start time as determined by dlmontepython and pymbar. Figure S2 was constructed using code available here: `https://gitlab.com/dl_monte/dlmontepython/-/blob/master/`

Table S2: Predicted $T_g$ values using average densities computed from the detection of steady-state until the end of the simulation, using the fitting method of Patrone *et al.* Dlmontepython and pymbar are used to determine the start time of a steady-state sample.

| dlmontepython | | | | | | |
|---|---|---|---|---|---|---|
| Protocol | Model **I** | Model **II** | Model **III** | Model **IV** | Model **V** | Model **VI** |
| Parallel | 437.2 ± 3.7 | 433.3 ± 4.8 | 446.8 ± 2.1 | 422.6 ± 2.6 | 540.1 ± 9.2 | 519.0 ± 2.8 |
| $\text{Parallel}_{\text{cold}}$ | 442.4 ± 2.3 | 433.6 ± 3.3 | 449.1 ± 3.1 | 423.4 ± 2.3 | 563.2 ± 9.2 | 531.3 ± 4.9 |
| pymbar | | | | | | |
| Protocol | Model **I** | Model **II** | Model **III** | Model **IV** | Model **V** | Model **VI** |
| Parallel | 435.8 ± 4.3 | 433.5 ± 3.3 | 445.6 ± 1.7 | 421.5 ± 3.0 | 539.0 ± 8.9 | 520.6 ± 4.9 |
| $\text{Parallel}_{\text{cold}}$ | 442.9 ± 2.1 | 433.4 ± 3.3 | 446.9 ± 2.2 | 425.1 ± 3.6 | 562.57 ± 8.2 | 531.0 ± 5.0 |

`dlmontepython/simtask/analysis.py`.

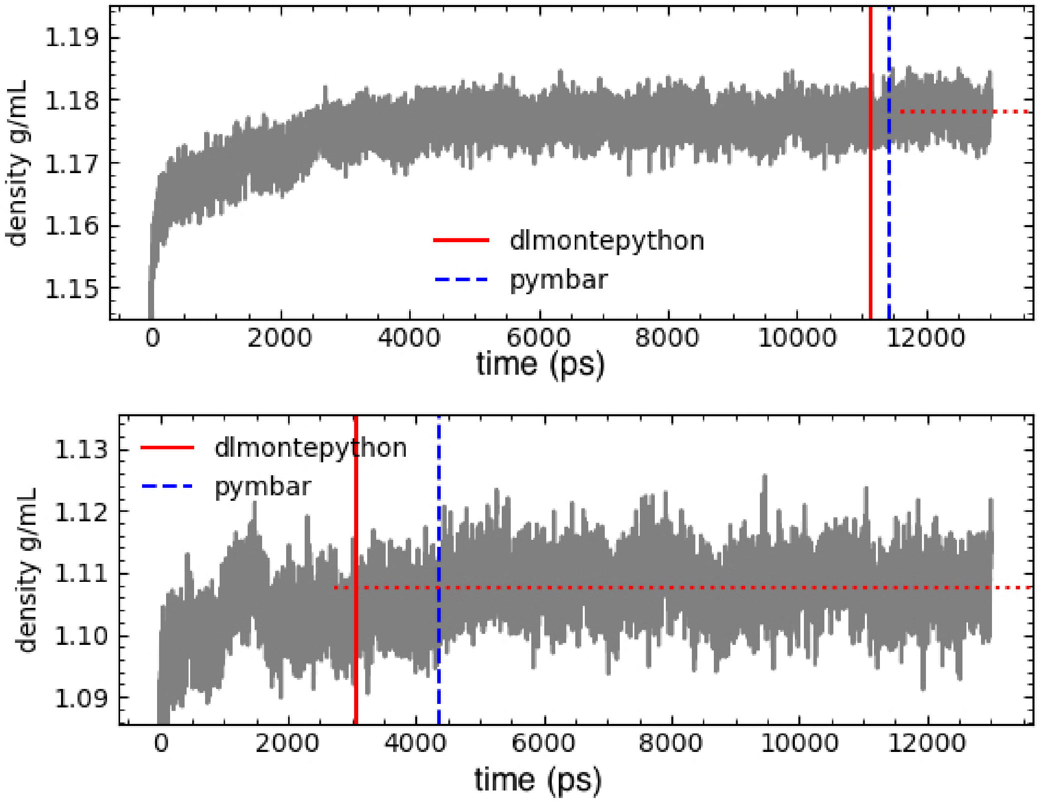


a)

b)

Figure S2: Time evolution of the density for a single selected replica of Model **I** using the parallel protocol. Solid red lines indicate the detection of steady-state according to the dlmontepython heuristic, blue dotted lines indicate steady-state according to the pymbar heuristic. a) 300K, b) 550K. Note, the system was first run for 13ns at 550K before being used as initial coordinates for the required temperature.

## 5 DETECTION OF STEADY-STATE / META-STABILITY FOR THE INITIAL HIGH-TEMPERATURE SIMULATIONS

In the computational workflow illustrated in Figure 2 of the paper, each resin system is initially run for 13ns at a high temperature, which is at least 100K greater than the experimental $T_g$. The final snapshot of these simulations is used as the initial coordinates for our workflows, shown in Figure 2. At these elevated temperatures, the additional mobility should be high enough for the cross-linked epoxy network to explore low-energy states. To ensure these systems had reached either equilibrium or long-lasting meta-stable states before the subsequent steps in the workflow, we used the dlmontepython and pymbar programmes to ensure that there was no drift in the density or potential energy at 13ns.

Plots of the density and potential energy for each resin system, along with the dlmontepython and pymbar determined steady-state (*i.e.* no detectable drift in values) are shown in Figure S3 and S4. These figures show that all systems reach stability at least 3ns before the end of the elevated temperature simulations.

The automatic detection of steady-state / meta-stability have also been used to ensure there is no drift in values at each temperature during the stepwise protocols. An illustration of the time evolution of the density of a selected replica and temperature is given in Figure S5 for the Stepwise_0.5ns protocol.

## 6 DISTRIBUTIONS OF $T_g$ VALUES USING THE LIN FITTING METHOD

In Figure S6 we show the distributions of the $T_g$ predictions using the Lin fitting method using bootstrapping analysis for the parallel, $\text{parallel}_{\text{cold}}$, stepwise_density_2ns and stepwise_Tg_2ns protocols. A normal distribution is especially clear for the distributions of $T_g$ for the stepwise_Tg_2ns protocol; this is to be expected as we use bootstrapped sampling of the $T_g$ computed for each stepwise replica and, as a result of the central-limit theorem, a Gaussian distribution is found. As is the case for the Patrone fitting method (Figure 7 in the

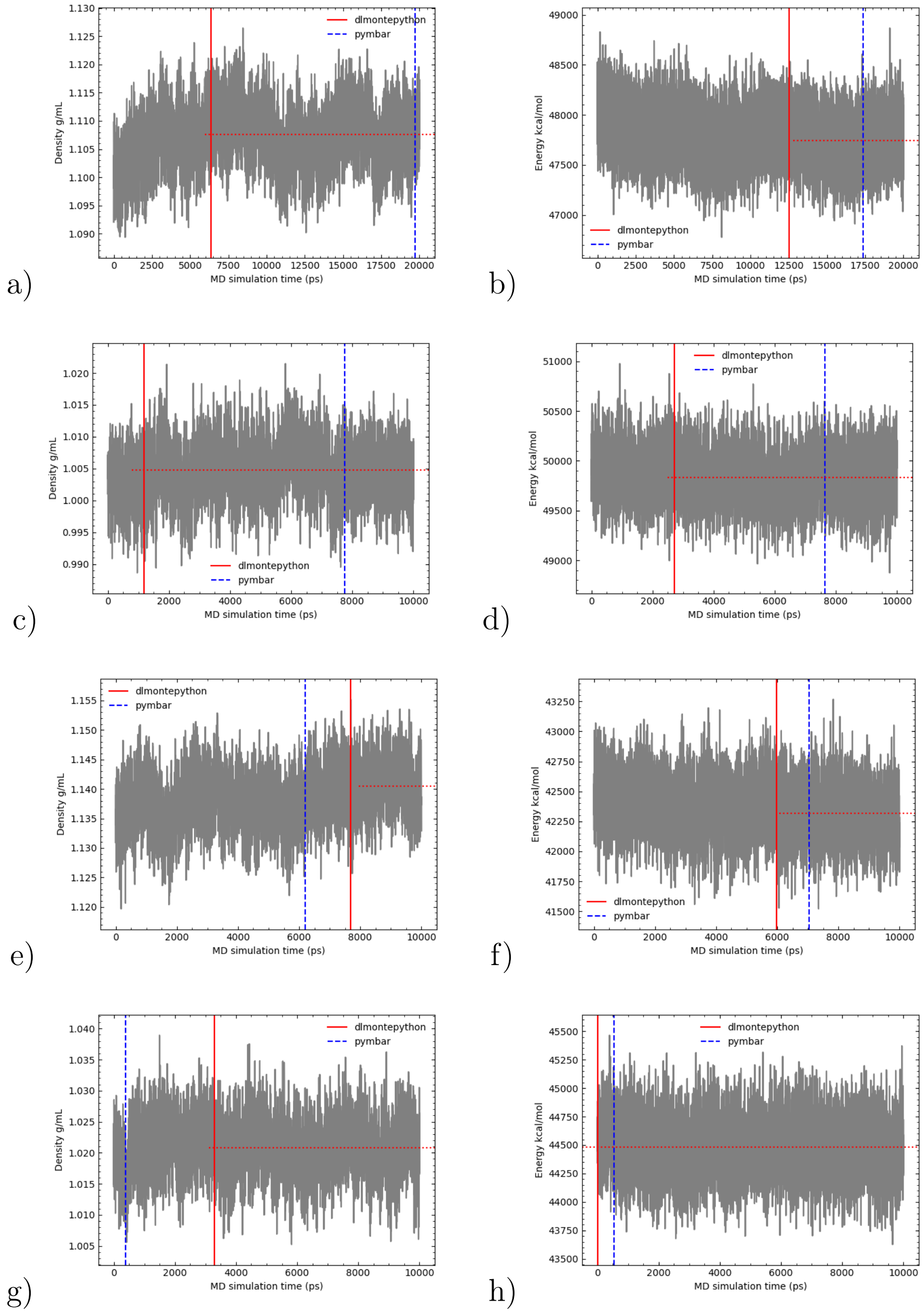


Figure S3: Time evolution of the density and potential for resin models **I-IV** using the parallel protocol of the initial high-temperature simulation at 550K. Solid red lines indicate the detection of a steady-state according to the dlmontepython heuristic, blue dotted lines indicate steady-state / meta-stability according to the pymbar heuristic. a), c), e) and g) are the evolution of the density for models **I-IV** respectively and b), d), f) and i) are the evolution of the potential energy.

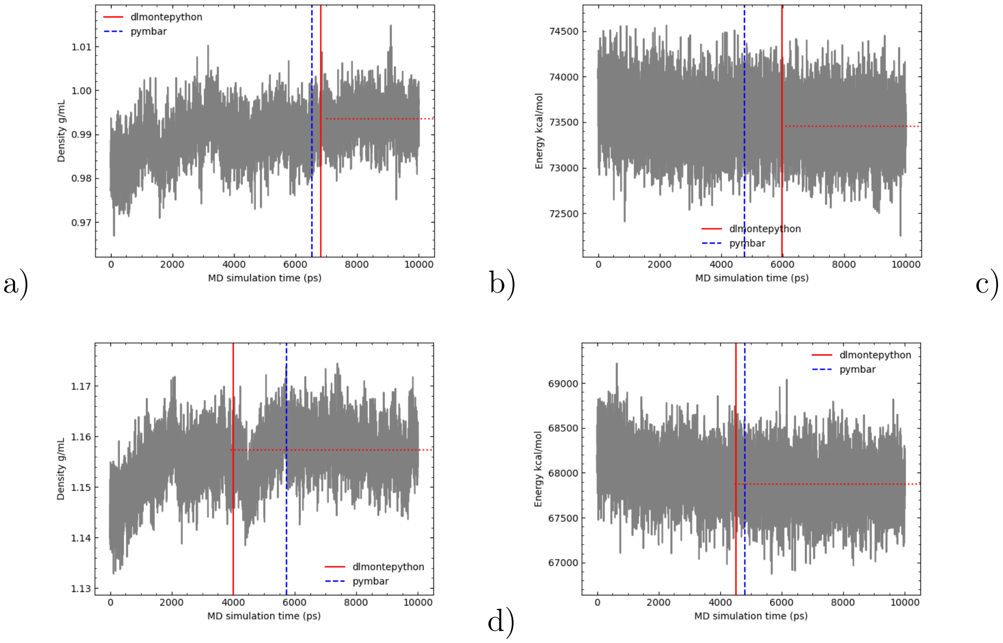


Figure S4: Time evolution of the density and potential for resin models **V** and **VI** of the initial simulation at 650K. Solid red lines indicate the detection of a steady-state according to the dlmontepython heuristic, blue dotted lines indicate steady-state according to the pymbar heuristic. a) and c) are the evolution of the density for models **V** and **VI** respectively, and b) and d) are the evolution of the potential energy.

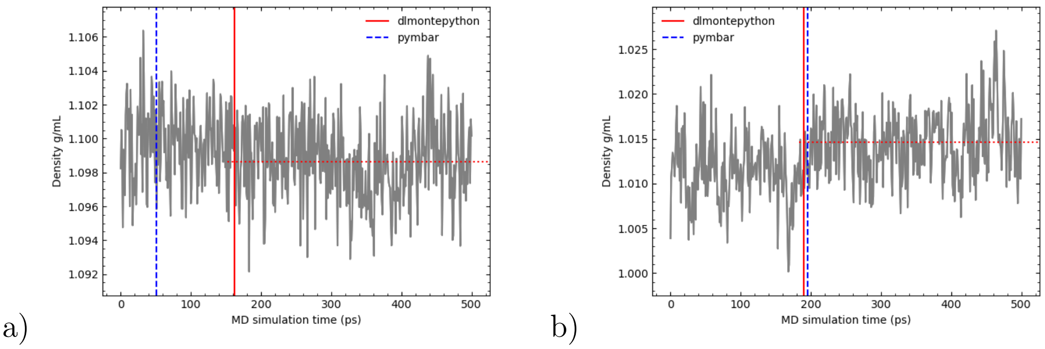


Figure S5: Time evolution of the density for resin model **II** using the Stepwise_0.5ns at a) 330K and b) 530K. Solid red lines indicate the detection of a steady-state / meta-stability according to the dlmontepython heuristic, blue dotted lines indicate steady-state / meta-stability according to the pymbar heuristic.

main paper), in the majority of the other cases, however, we find multimodal distributions.

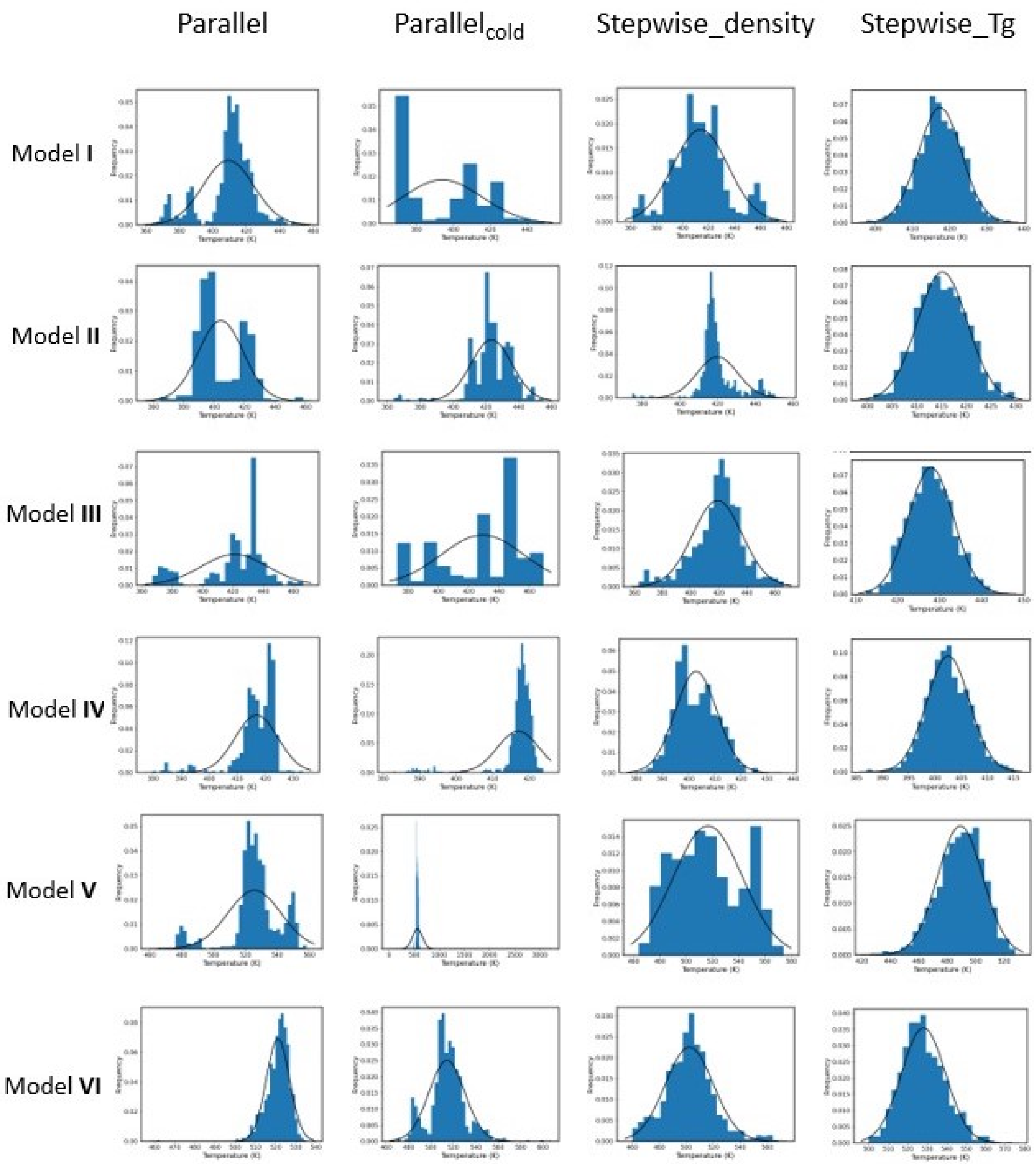


Figure S6: The distributions of $T_g$ from the bootstrapped sampling of densities using the Lin fitting method. The columns are the different protocols, as follows: 1. Parallel; 2. Parallel$_{\text{cold}}$; 3 Stepwise_density_2ns; 4. Stepwise_Tg_2ns. The systems are: a)DGEBA-44DDS (model **I**), b) DGEBA-MDEA (model **II**), c) DGEBF-44DDS (model **III**), d) DGEBF-MDEA (model **IV**), e) TGPAP-MDEA (model **V**), f) TGPAP-44DDS (model **VI**). A fitted normal distribution is shown for each plot. For the majority of distributions, a multimodal distribution is observed.

## 7 DISTRIBUTION OF $T_g$ VALUES COMPUTED USING THE STEPWISE PROTOCOLS WITHOUT BOOTSTRAPPING

In Figures S7 and S8 we show distributions of computed Tg values from the 20 replicas of the Stepwise_Tg_2ns protocol using the fitting methods of Patrone *et al.* and Lin *et al.*

respectively. No bootrapping was used to compute the distributions; Figures S7 and S8. It can be clearly seen in Figures S7 and S8 that the distributions are non-normal.

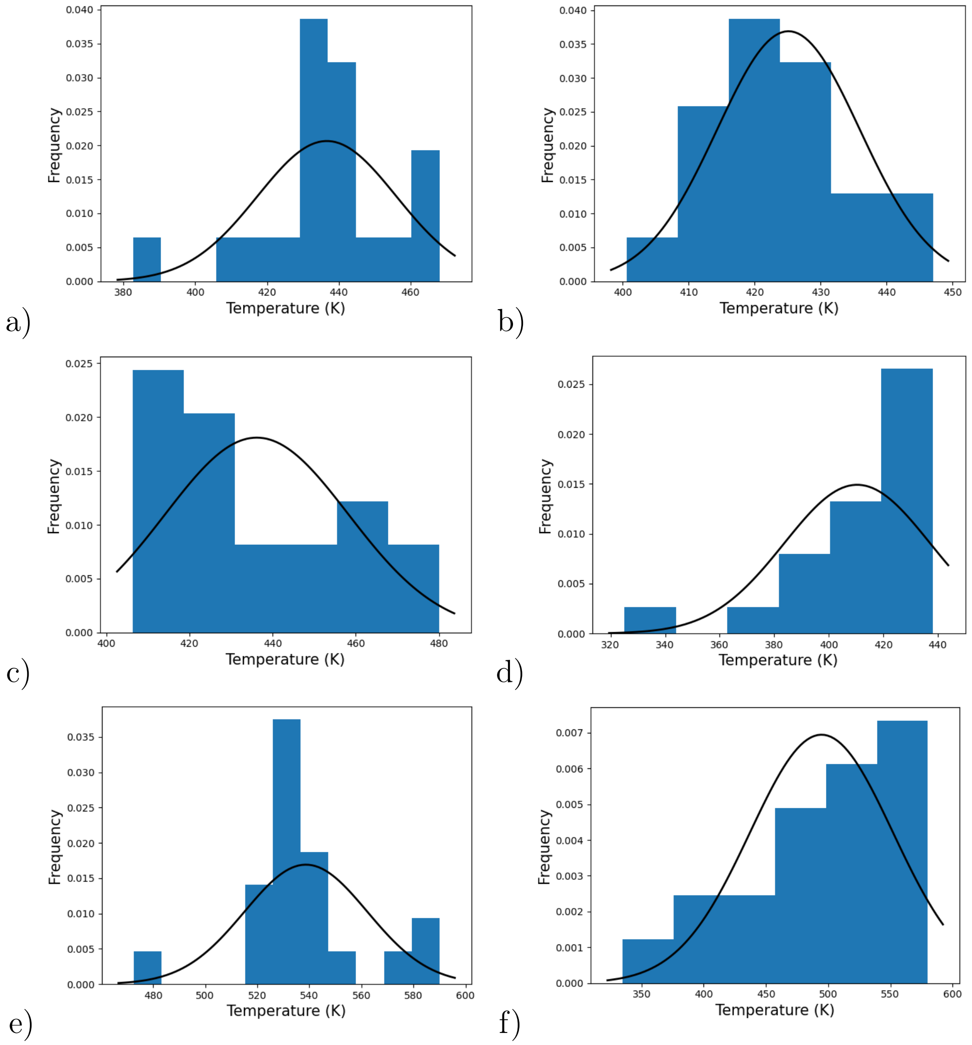


Figure S7: Distributions of the 20 $T_g$ computed using the Stepwise_Tg_2ns protocol and the fitting method of Patrone *et al.*: a) system **I**, b) system **II**, c) system **III**, d) system **IV**, e) system **V**, f) system **VI**. The black lines show a fit to a normal distribution.

## 8 DEPENDENCE OF ESTIMATED $T_g$ ON THE SIZE OF THE ENSEMBLE

In Figure 4 in the main paper, we show the 50% percentile values and 95% confidence intervals for $T_g$ predictions using bootstrapping for all models as a function of number of members of the ensemble, using the fitting method of Patrone *et al.* In Tables S3, S4, S5

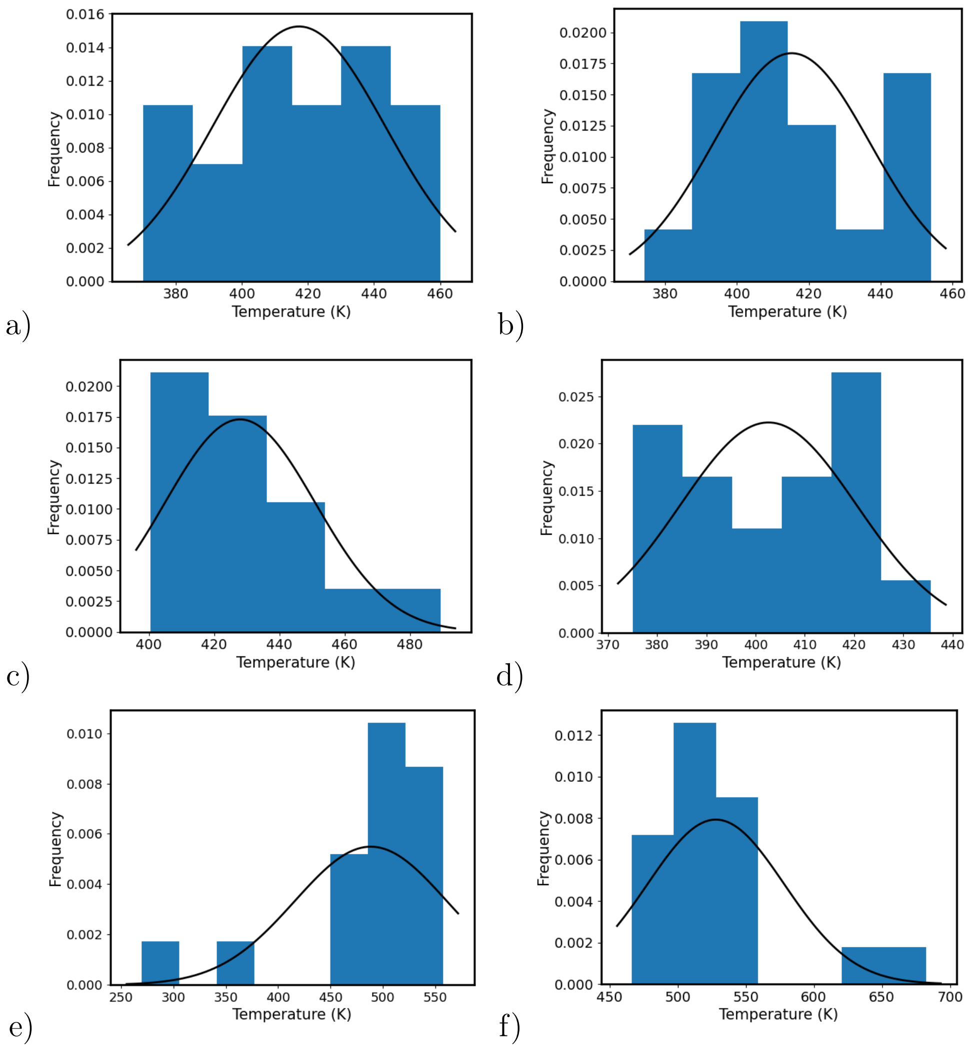


Figure S8: Distributions of the 20 $T_g$ computed using the Stepwise_Tg_2ns protocol and the fitting method of Lin *et al.*: a) system **I**, b) system **II**, c) system **III**, d) system **IV**, e) system **V**, f) system **VI**. The black lines show a fit to a normal distribution.

and S6 we list the 50% percentiles and 95% confidence intervals used to construct Figure 4 in the main paper for the various protocols and models in our study. Tables S3 - S6 illustrate the $N^{-0.5}$ scaling in 95% confidence interval with number of replicas in the ensemble.

## 9 DEPENDENCE OF ESTIMATED $T_g$ WITH LENGTH OF SIMULATION

In Figure 6 in the main paper, we present the change in the 95% confidence interval of predicted $T_g$ values as a function of total MD simulation time (upto 13ns), averaged over all

Table S3: Dependence of 95% confidence interval on ensemble size using the fitting method of Patrone for Models **I-III** for parallel protocols.

| System | Protocol | number of replicas | 50% percentile (K) | 95% confidence interval (K) |
|---|---|---|---|---|
| Model **I** | Parallel | 1 | 439.0 | 57.6 |
| | | 5 | 437.9 | 25.4 |
| | | 10 | 440.5 | 15.6 |
| | | 15 | 440.9 | 13.5 |
| | | 20 | 440.9 | 12.3 |
| Model **I** | $\text{Parallel}_{\text{cold}}$ | 1 | 441.0 | 47.1 |
| | | 5 | 441.7 | 16.2 |
| | | 10 | 441.9 | 12.5 |
| | | 15 | 441.8 | 10.8 |
| | | 20 | 441.8 | 9.8 |
| Model **II** | Parallel | 1 | 433.4 | 45.7 |
| | | 5 | 432.2 | 22.7 |
| | | 10 | 432.1 | 19.7 |
| | | 15 | 432.1 | 17.9 |
| | | 20 | 432.0 | 17.5 |
| Model **II** | $\text{Parallel}_{\text{cold}}$ | 1 | 434.0 | 38.7 |
| | | 5 | 433.0 | 21.2 |
| | | 10 | 432.9 | 17.4 |
| | | 15 | 432.9 | 15.9 |
| | | 20 | 432.8 | 11.2 |
| Model **III** | Parallel | 1 | 445.7 | 46.7 |
| | | 5 | 446.3 | 21.4 |
| | | 10 | 446.3 | 12.3 |
| | | 15 | 446.4 | 10.2 |
| | | 20 | 446.4 | 9.5 |
| Model **III** | $\text{Parallel}_{\text{cold}}$ | 1 | 447.1 | 56.3 |
| | | 5 | 447.4 | 20.2 |
| | | 10 | 447.4 | 13.0 |
| | | 15 | 447.4 | 11.6 |
| | | 20 | 447.5 | 10.4 |

resin models for 20 replicas on the duration of the MD simulation and the time-frame used to compute the average density of the system. In Figure S9, we show that all the predicted $T_g$ for each resin model are within the average 95% confidence interval for the parallel protocol (red error bar), irrespective of duration and start point of density data collection. Therefore, a minimum of 4ns of burn-in time and 2ns of data collection is sufficient for the parallel

Table S4: Dependence of 95% confidence interval on ensemble size using the fitting method of Patrone for models **IV** - **VI** for parallel protocols

| System | Protocol | number of replicas | 50% percentile (K) | 95% confidence interval (K) |
|---|---|---|---|---|
| Model **IV** | Parallel | 1 | 424.3 | 43.4 |
| | | 5 | 422.2 | 25.7 |
| | | 10 | 422.1 | 20.6 |
| | | 15 | 421.9 | 18.7 |
| | | 20 | 421.9 | 18.3 |
| Model **IV** | $\text{Parallel}_{\text{cold}}$ | 1 | 425.6 | 36.5 |
| | | 5 | 423.9 | 20.6 |
| | | 10 | 423.4 | 12.3 |
| | | 15 | 423.5 | 11.1 |
| | | 20 | 423.5 | 10.5 |
| Model **V** | Parallel | 1 | 539.8 | 73.7 |
| | | 5 | 538.0 | 33.9 |
| | | 10 | 538.1 | 25.1 |
| | | 15 | 538.0 | 20.6 |
| | | 20 | 538.0 | 24.6 |
| Model **V** | $\text{Parallel}_{\text{cold}}$ | 1 | 554.4 | 101.7 |
| | | 5 | 560.0 | 64.6 |
| | | 10 | 560.0 | 53.4 |
| | | 15 | 560.0 | 41.0 |
| | | 20 | 559.5 | 35.4 |
| Model **VI** | Parallel | 1 | 525.3 | 65.2 |
| | | 5 | 521.5 | 29.3 |
| | | 10 | 521.0 | 22.2 |
| | | 15 | 520.9 | 19.5 |
| | | 20 | 520.6 | 18.3 |
| Model **VI** | $\text{Parallel}_{\text{cold}}$ | 1 | 531.9 | 76.3 |
| | | 5 | 532.4 | 36.5 |
| | | 10 | 531.6 | 29.9 |
| | | 15 | 532.1 | 21.2 |
| | | 20 | 532.0 | 18.7 |

protocol, as Figure 6 in the main paper shows that the 95% confidence interval is close to its smallest value at this point; there is no significant change in either predicted $T_g$ values or 95% confidence intervals if simulated for longer. For the $\text{Parallel}_{\text{cold}}$ protocol, the situation is the same for simulations longer than 5ns, except for a few outliers for Model **IV**. We can therefore conclude that for the $\text{Parallel}_{\text{cold}}$ protocol, 2ns of burn-in and 5ns of data collection

Table S5: Dependence of 95% confidence interval on ensemble size using the fitting method of Patrone for Models **I-III** for stepwise protocols.

| System | Protocol | number of replicas | 50% percentile (K) | 95% confidence interval (K) |
|---|---|---|---|---|
| | | 1 | 440.0 | 76.6 |
| | | 5 | 439.2 | 35.4 |
| Model **I** | Stepwise_density_2ns | 10 | 439.1 | 17.2 |
| | | 15 | 439.0 | 15.0 |
| | | 20 | 439.3 | 14.3 |
| | | 1 | 438.1 | 85.3 |
| | | 5 | 437.5 | 33.6 |
| Model **I** | Stepwise_Tg_2ns | 10 | 436.8 | 24.1 |
| | | 15 | 436.7 | 20.0 |
| | | 20 | 436.7 | 17.3 |
| | | 1 | 430.0 | 46.1 |
| | | 5 | 427.3 | 20.8 |
| Model **II** | Stepwise_density_2ns | 10 | 425.5 | 13.6 |
| | | 15 | 425.3 | 11.7 |
| | | 20 | 424.7 | 10.8 |
| | | 1 | 423.5 | 46.4 |
| | | 5 | 425.5 | 18.8 |
| Model **I** | Stepwise_Tg_2ns | 10 | 425.7 | 14.2 |
| | | 15 | 425.8 | 11.2 |
| | | 20 | 425.7 | 9.8 |
| | | 1 | 433.9 | 70.3 |
| | | 5 | 434.5 | 34.8 |
| Model **III** | Stepwise_density_2ns | 10 | 434.5 | 24.9 |
| | | 15 | 434.1 | 20.8 |
| | | 20 | 434.1 | 15.4 |
| | | 1 | 432.7 | 73.8 |
| | | 5 | 437.0 | 39.2 |
| Model **III** | Stepwise_Tg_2ns | 10 | 437.3 | 27.8 |
| | | 15 | 437.4 | 23.1 |
| | | 20 | 437.6 | 20.6 |

are sufficient.

## 10 MOMENTS OF DENSITY DISTRIBUTIONS

In Figure 7 in the main paper, we find multimodal distributions of predicted $T_g$ values. It is instructive to look at the density distribution at each temperature point across the ensembles

Table S6: Dependence of 95% confidence interval on ensemble size using the fitting method of Patrone for models **IV**-**VI** for stepwise protocols.

| System | Protocol | number of replicas | 50% percentile (K) | 95% confidence interval (K) |
|---|---|---|---|---|
| Model **IV** | Stepwise_density_2ns | 1 | 412.6 | 53.2 |
| | | 5 | 411.1 | 28.6 |
| | | 10 | 411.0 | 21.1 |
| | | 15 | 410.8 | 19.3 |
| | | 20 | 410.9 | 18.8 |
| Model **IV** | Stepwise_Tg_2ns | 1 | 423.0 | 60.5 |
| | | 5 | 414.5 | 32.8 |
| | | 10 | 414.0 | 23.5 |
| | | 15 | 413.4 | 18.3 |
| | | 20 | 413.4 | 16.1 |
| Model **V** | Stepwise_density_2ns | 1 | 538.1 | 110.0 |
| | | 5 | 537.5 | 63.9 |
| | | 10 | 536.7 | 45.6 |
| | | 15 | 536.9 | 33.1 |
| | | 20 | 536.5 | 29.0 |
| Model **V** | Stepwise_Tg_2ns | 1 | 538.7 | 117.4 |
| | | 5 | 540.6 | 42.8 |
| | | 10 | 540.6 | 33.0 |
| | | 15 | 540.7 | 26.8 |
| | | 20 | 540.4 | 22.0 |
| Model **VI** | Stepwise_density_2ns | 1 | 520.0 | 112.7 |
| | | 5 | 513.0 | 68.6 |
| | | 10 | 513.0 | 56.6 |
| | | 15 | 512.7 | 56.1 |
| | | 20 | 513.1 | 43.8 |
| Model **VI** | Stepwise_Tg_2ns | 1 | 509.1 | 215.1 |
| | | 5 | 497.7 | 90.3 |
| | | 10 | 495.7 | 65.7 |
| | | 15 | 494.6 | 58.0 |
| | | 20 | 495.6 | 46.8 |

to determine why we observe these multimodal distributions for $T_g$. In Table S7, we show the standard deviation, skew and excess kurtosis for the density distributions averaged across all temperatures for all resins and protocols, averaged over 12-13ns for the parallel protocols, 1-2ns and 0.1-0.5ns for stepwise_2ns and stepwise_0.5ns protocols respectively. We find that the average excess kurtosis for the density distributions is negative. To understand why

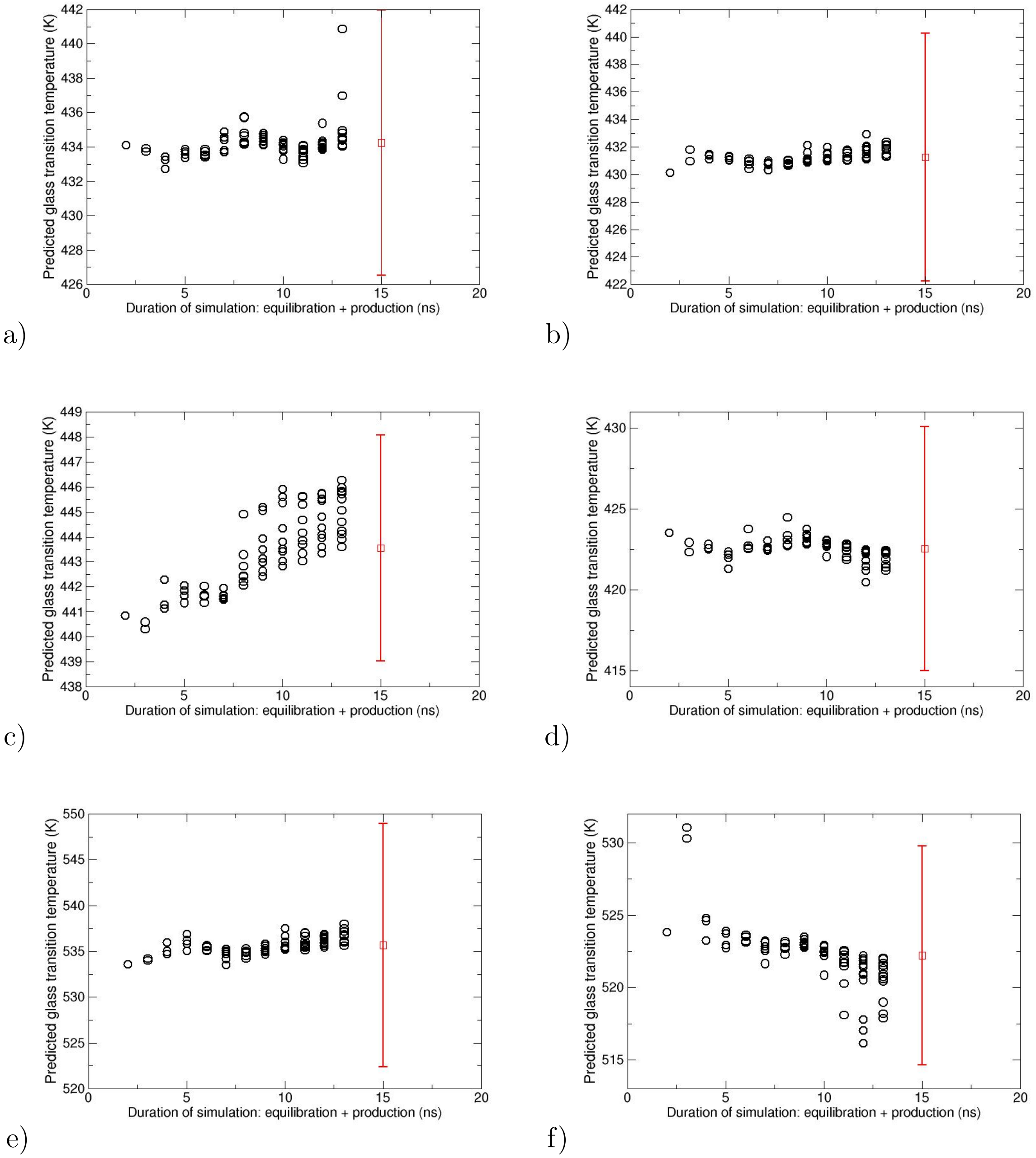


Figure S9: Predicted $T_g$ for each resin model as a function of the sum of burn-in time and data collection time (black circles) compared to the $T_g$ and 95% confidence interval averaged over all simulation durations (red error bar) for the parallel protocol, using the fitting method of Patrone with 20 replicas in the ensemble. The resin models are: a) **I**, b) **II**, c) **III**, d) **IV**, e) **V**, f) **VI**.

this is the case, in Figure S11 we examine quantile-quantile (Q-Q) plots for a selection of systems, protocols and temperature points and their corresponding histograms. Figure S11

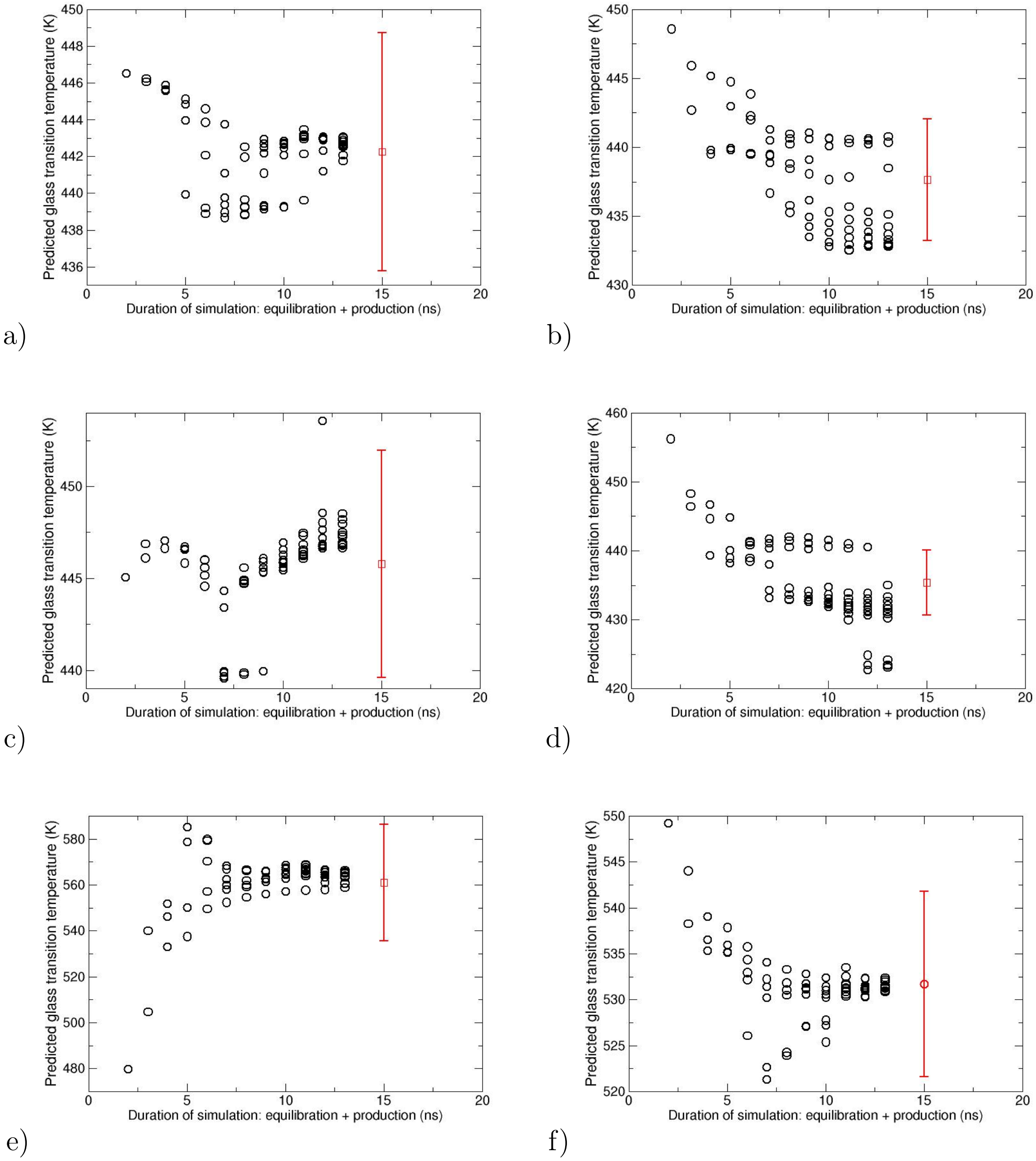


Figure S10: Predicted $T_g$ for each resin model as function of the sum of burn-in time and data collection time (black circles) compared to the $T_g$ and 95% confidence interval averaged over all simulation durations (red error bar) for the parallel$_{\text{cold}}$ protocol, using the fitting method of Patrone with 20 replicas in the ensemble. The resin models are: a) **I**, b) **II**, c) **III**, d) **IV**, e) **V**, f) **VI**.

shows that, for the selected densities, the Q-Q plot has a s-like curve. The corresponding histograms are shown in Figure S11, where the distribution is clearly bi- or multi-modal with

a significant separation between modes; this explains the thin-tails and the general s-shape of the Q-Q plots. As the density distributions are often multimodal, with a wide distribution of values, the distributions possess a negative excess kurtosis. As we show in the main paper, the wider the distribution of densities at each temperature point, the higher the likelihood that the solutions to the fitting method of Patrone will cluster to different predicted $T_g$ values, leading to multi-modal distributions.

Table S7: Moments of the density distributions averaged across all temperatures.

| Resin | Scenario | Mean St. dev | Mean Skew | RMS Skew | Mean kurtosis | RMS Kurtosis |
|---|---|---|---|---|---|---|
| Model **I** | Parallel | 0.00181 | -0.04 | 0.52 | -0.38 | 0.97 |
| | $\text{Parallel}_{\text{cold}}$ | 0.00150 | -0.17 | 0.47 | -0.24 | 0.94 |
| | Stepwise_density_2ns | 0.00195 | -0.52 | 0.71 | 0.28 | 0.98 |
| | Stepwise_density_0.5ns | 0.00187 | -0.14 | 0.43 | -0.50 | 0.81 |
| Model **II** | Parallel | 0.00173 | 0.12 | 0.47 | -0.32 | 0.84 |
| | $\text{Parallel}_{\text{cold}}$ | 0.00156 | 0.01 | 0.43 | -0.41 | 0.69 |
| | Stepwise_density_2ns | 0.00157 | 0.16 | 0.40 | -0.41 | 0.62 |
| | Stepwise_density_0.5ns | 0.00178 | 0.04 | 0.37 | -0.57 | 0.84 |
| Model **III** | Parallel | 0.00180 | 0.17 | 0.50 | -0.25 | 0.98 |
| | $\text{Parallel}_{\text{cold}}$ | 0.00165 | 0.01 | 0.45 | -0.45 | 0.86 |
| | Stepwise_density_2ns | 0.00210 | -0.02 | 0.47 | -0.50 | 0.87 |
| | Stepwise_density_0.5ns | 0.00201 | -0.50 | 0.66 | -0.16 | 0.81 |
| Model **IV** | Parallel | 0.00161 | 0.05 | 0.40 | -0.65 | 0.81 |
| | $\text{Parallel}_{\text{cold}}$ | 0.00158 | -0.15 | 0.47 | -0.61 | 0.86 |
| | Stepwise_density_2ns | 0.00176 | -0.07 | 0.43 | -0.58 | 0.90 |
| | Stepwise_density_0.5ns | 0.00166 | 0.05 | 0.70 | 0.08 | 1.21 |
| Model **V** | Parallel | 0.00199 | -0.12 | 0.47 | -0.37 | 0.75 |
| | $\text{Parallel}_{\text{cold}}$ | 0.00213 | -0.05 | 0.69 | -0.04 | 1.08 |
| | Stepwise_density_2ns | 0.00233 | 0.20 | 0.46 | -0.41 | 0.72 |
| Model **VI** | Parallel | 0.00189 | -0.11 | 0.48 | -0.19 | 0.85 |
| | $\text{Parallel}_{\text{cold}}$ | 0.00205 | 0.10 | 0.52 | -0.35 | 0.98 |
| | Stepwise_density_2ns | 0.00205 | -0.02 | 0.50 | -0.32 | 0.74 |
| | Stepwise_density_0.5ns | 0.00230 | -0.28 | 0.61 | -0.25 | 0.70 |

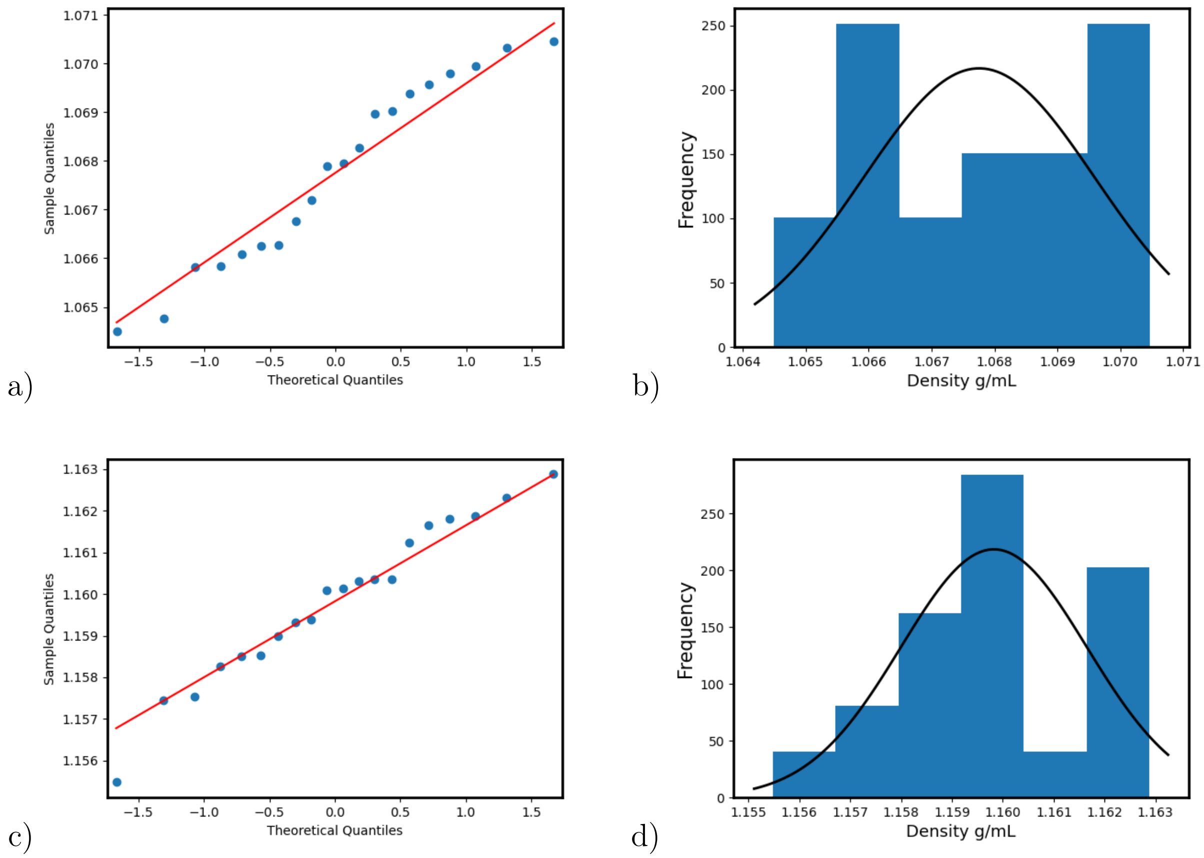


Figure S11: Selected quantile-quantile (Q-Q) plot and histograms compared to a normal distribution. a) Q-Q plot for the DGEBF-MDEA (model **IV**) system using the Stepwise_density_2ns scenario at 470K (red line is the normal distribution), which shows a multimodal distribution for $T_g$ in the corresponding probability distribution (b)). c) (Q-Q) plot for the DGEBF-MDEA (model **IV**) system using the $Parallel_{cold}$ protocol at 410K and d) the corresponding probability distribution, which shows a similar multi-modal distribution.

## 11 COMPARISON OF THE STANDARD DEVIATIONS OF DENSITY DISTRIBUTION ACROSS ENSEMBLE AND WITHIN SINGLE SIMULATIONS

In Figure S12, we show a comparison between the standard deviation in mean densities across the 20 replicas in our ensemble, compared to the average standard deviation in density in a single replica, determined using block averaging.

The block averaged densities were computed using the dlmontepython programme[7]. The correlation time, as computed by dlmontepython, is used to inform the block size, with each

block defined as 4 times the correlation time. A set of densities is compiled for each replica, corresponding to each block after steady-state is determined to start. The average standard deviation of these blocks across all replicas and models is shown in Figure S12. For details on how dlmontepython calculates the correlation time, please see Reference 7.

Figure S12 shows that the block-averaged standard deviation of densities increases linearly as temperature is increased. However, the standard deviation in mean densities across the ensemble is relatively constant across temperatures for both parallel and $\text{parallel}_{\text{cold}}$ protocols. A higher temperatures, the two standard deviations are comparable, indicating that individual replicas can sample multiple metastable states, but at lower temperatures (below the glass transition temperature) the states sampled by single replicas are much more restricted and the standard deviation in mean densities across the ensemble is much higher. This indicates why using ensembles of MD simulations is so essential: due to the divergence in trajectories caused by the use of random seeds in the MD code, we can sample many more microstates than using single MD simulations (even if the latter are run for much longer)[10,11].

To illustrate the wider distribution of densities produced from an ensemble of replica simulations compared to the variation in density of single simulations, we have plotted in Figure S13 the standard deviation in density computed by block averaging compared to ensemble standard deviation for selected models at 300K using the parallel protocol.

## 12 CORRELATIONS BETWEEN DENSITY AND OTHER PROPERTIES

In section 11 a wide distribution of densities are found within each ensemble, which are stable on the timescale of the MD simulations. We have analysed the correlation between the density and energetic properties of each replica, to gain insight into the factors that cause the distribution of densities. In Tables S8 and S9, we show the Pearson correlation coefficient for the variation of the density with the potential, van der Waals and coulombic energies of the system, averaged over all temperatures. Tables S8 and S9 clearly show that higher densities are correlated with lower (*i.e.* more attractive) van der Waals interactions.

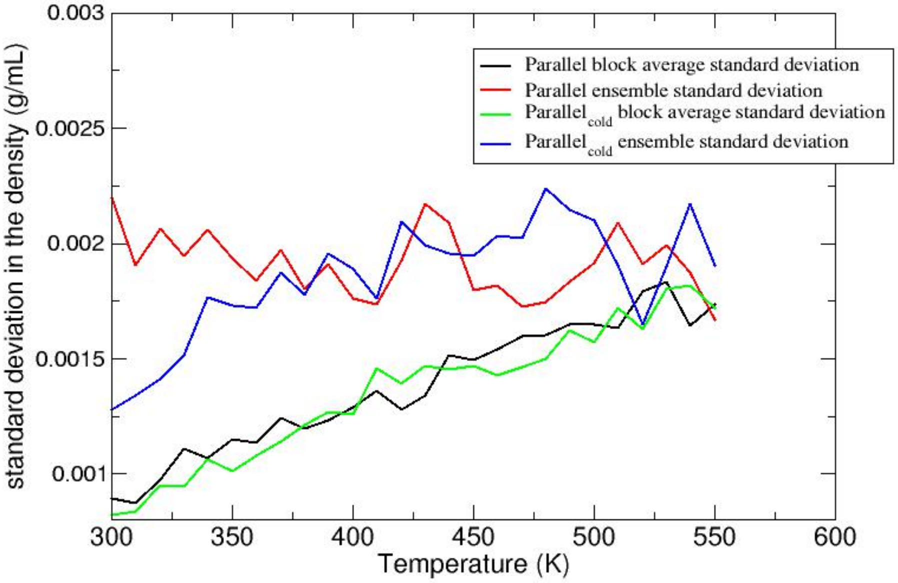


Figure S12: Comparison of the standard deviation in densities computed across the ensemble using the mean density of each replica (red for the parallel protocol, blue for the parallel$_{\text{cold}}$ protocol) and the average standard deviation computed using block-averaging within a single replica (black for the parallel protocol, green for the parallel$_{\text{cold}}$ protocol).

This correlation is not shown for the coulombic energies, and is therefore much reduced for the overall potential energy. This indicates that increasing the van der Waals interactions leads to more effective packing of the cross-linked resin.

To further illustrate the correlation between van der Waals interactions and the density of the system, in Figure S14, we show the average density and van der waals interaction energies for each replica in the ensemble for model **II** at 300K using the stepwise_2ns protocol; the correlation can clearly be seen.

Table S8: Pearson correlation coefficients for the correlation between density and energetic properties of the resin system for the $\text{Parallel}_{\text{hot}}$ protocol, averaged over all temperatures.

| Resin | van der Waals energy | potential energy | coulomb energy |
|---|---|---|---|
| **I** | -0.87 | -0.58 | -0.05 |
| **II** | -0.88 | -0.72 | -0.11 |
| **III** | -0.89 | -0.57 | -0.06 |
| **IV** | -0.86 | -0.70 | -0.17 |
| **V** | -0.90 | -0.71 | -0.23 |
| **VI** | -0.85 | -0.60 | -0.18 |

Table S9: Pearson correlation coefficients for the correlation between density and energetic properties of the resin system for the Stepwise_2ns protocol, averaged over all temperatures.

| Resin | van der Waals energy | potential energy | coulomb energy |
|---|---|---|---|
| **I** | -0.88 | -0.48 | -0.07 |
| **II** | -0.84 | -0.59 | 0.03 |
| **III** | -0.88 | -0.71 | -0.12 |
| **IV** | -0.84 | -0.73 | -0.28 |
| **V** | -0.88 | -0.71 | -0.25 |
| **VI** | -0.86 | -0.62 | -0.07 |

## 13 DETERMINING THE GLASS TRANSITION TEMPERATURE USING SEGMENTAL RELAXATION TIMES

As a comparison to computing the glass transition using the density-temperature curve, we have investigated estimating the glass transition temperature via bond autocorrelation functions for the epoxy-resin networks[12].

To obtain the segmental relaxation times, we examined the 2nd order Legendre polynomial of the time autocorrelation function ($P_2(t)$) of selected bond vectors, *i.e.* the orientational mobility of the bonds in the system:

$$P_2(t) = \frac{1}{2}(3\langle \mathbf{u}(0) \cdot \mathbf{u}(t) \rangle^2 - 1) \tag{1}$$

where the brackets denote averaging over all bonds in the ensemble and $\mathbf{u}(t)$ is the bond vector at time t.

The computed time correlation functions were fitted to exponential decays or stretched

exponential functions to extract relaxation times:

$$P_2(t) \sim \exp\left[-\left(\frac{t}{\tau_{\text{corr}}}\right)^{\beta}\right] \quad (2)$$

where $\tau_{\text{corr}}$ is the correlation time and $\beta$ is the stretching exponent.

The segment relaxation time $\tau_c$ can be computed from the segment characteristic correlation time $\tau_{\text{corr}}$ and the stretching exponent $\beta$ using the following relationship:

$$\tau_c = \tau_{\text{corr}} \cdot \Gamma\left(\frac{1}{\beta}\right) \quad (3)$$

where $\tau_c$ is the segment relaxation time, $\tau_{\text{corr}}$ is the characteristic correlation time, $\beta$ is the stretching exponent (typically $0 < \beta \leq 1$) and $\Gamma(x)$ is the Gamma function.

Plotting $\tau_c$ as a function of temperature, we expect to see a shift from Arrhenius behaviour at the glass transition temperature, where relaxation times dramatically increase. This behaviour is described using the Vogel-Fulcher-Tammann (VFT) equation, given by:

$$\tau_c = \tau_0 \exp\left(\frac{D}{T - T_0}\right) \quad (4)$$

where: $\tau$ is the relaxation time, $\tau_0$ is a pre-exponential factor, $D$ is a characteristic constant, $T$ is the temperature, $T_0$ is the Vogel temperature.

In the context of the glass transition, the Arrhenius behavior implies that at high temperatures, where $k_BT \gg E_a$, the material behaves like a normal liquid or polymer, with rapid molecular motions. As the temperature decreases and $k_BT$ becomes comparable to $E_a$, the material begins to exhibit glassy behavior, with slower dynamics and structural relaxation.

In Figure S15, we show the correlation times as a function of temperature for the phenyl carbon-hydrogen bonds for the resin models from the Stepwise_2ns protocol. We find that for bond-vectors involving the phenyl rings, there is a detectable shift from Arrhenius to non-Arrhenius (VFT) behaviour. We have listed in Table S10 the glass temperature determined as the start of deviation from Arrhenius behaviour. It should be noted there is

ambiguity in determining glass transition in this manner, as it relies on user identification of the abrupt change in the correlation times. Comparing with the $T_g$ derived from the density-temperature behaviour (Table 3 in the main paper), the $T_g$ values are in general lower (although within 15K of those predicted using the Lin method (Table **??**). As Figure S15 show, the transition from Arrhenius to VFT behaviour is gradual, and hence we relate the values in Table S10 as indicating the start of the glass transition region.

For backbone atoms such as the carbon-oxygen bonds (Figure S16), we find the orientation relaxation times at low temperatures are either consistent with or higher than Arrhenius behaviour. This indicates that it is the motion of the phenyl rings that is the characteristic segmental motion associated with the glass transition[13].

Table S10: Glass transition temperatures estimated from the segmental relaxation times in Figures S15 and S16. The glass transition temperatures listed in this figure correspond to the dashed vertical lines in Figures S15 and S16.

| Resin | glass transition temperature (K) |
|---|---|
| **I** | 405 |
| **II** | 399 |
| **III** | 408 |
| **IV** | 392 |
| **V** | 559 |
| **VI** | 541 |

## 14 DENSITY ANALYSIS OF MULTIMODAL $T_g$ DISTRIBUTIONS.

To examine the difference in density distributions in the density-temperature curve that can propagate to create multi-modal $T_g$ distributions, we have compared the density-temperature curves that leads to a high $T_g$ peak and compared them to those that produce a low $T_g$ peak.

The Stepwise_density_2ns scenario for model **II** is a good example: the $T_g$ distribution has two distinct distributions (Figure 7 in the main paper). We have separated the density-temperature curves dervied from the bootstrapping of the densities into those that result in a $T_g$ being in either the low or high peaks. We have examined the difference between the

average densities of the "high" and "low" $T_g$ density-temperature curves to examine common features. In Figure S17, we show that "higher" $T_g$s have higher densities at temperatures at and below the glass transition and lower densities above the glass transition. A schematic is shown in Figure S17.

We have found that this is a general observation for all scenarios and resin models: higher $T_g$s have densities which are higher at temperatures up to the glass transition and have lower density at temperatures higher than $T_g$. We have previously shown that there is a high correlation between density and favourable van der Waals interactions. We can therefore postulate that higher $T_g$ peaks are due to transitions from densities with more favourable ensemble averaged van der Waals interactions at and below the glass transition to less favourable van der Waals interactions at temperatures above the glass transition.

## 15 $T_g$ DERIVED FROM THE DIFFUSION COEFFICIENTS

As an additional comparison we have computed $T_g$ using the mean-squared displacement of the atoms. For each replica, the average mean-squared displacement (MSD) as a function of time is calculated over all atoms at each temperature. The position of the atoms are recorded every 0.1ns. A linear regression of the MSD, ignoring the first 0.2ns of simulation, is performed. The gradient of the linear fit is 6 times the diffusion coefficient.

The MSD averaged over all atoms at time $t$ is given by:

$$\langle \overline{r^2(t)} \rangle = \frac{1}{N} \sum_{i=1}^{N} \langle [\mathbf{r}_i(t) - \mathbf{r}_i(0)]^2 \rangle \tag{5}$$

where $N$ is the total number of atoms, $\mathbf{r}_i(t)$ is the position of the $i$-th atom at time $t$ and $\mathbf{r}_i(0)$ is the initial position of the $i$-th atom.

The equation that relates MSD to the diffusion coefficient $D$ is given by:

$$\langle r^2(t) \rangle = 2dDt \tag{6}$$

where $\langle r^2(t) \rangle$ is the mean squared displacement at time $t$, $D$ is the diffusion coefficient and $d$ is the dimensionality of the system.

The simulations reported here are in three-dimensional space ($d = 3$), therefore equation 6 becomes:

$$\langle r^2(t) \rangle = 6Dt$$

By plotting the diffusion coefficients as a function of temperature, we can use the same fitting techniques as we have demonstrated for the density-temperature behaviour to determine $T_g$.

Predicted $T_g$ values using the fitting method of Patrone are shown in Table S11. The standard deviations reported are across the ensemble. The large standard deviations indicate the large variation in values for the ensemble, which indicates the statistical uncertainty related to fitting the mean-squared displacement at each temperature point before subsequently fitting to determine $T_g$. However, it can be seen that the derived $T_g$ are within error of the $T_g$ computed from the density-temperature behaviour (Table 3 in the main paper).

Table S11: Glass transition temperatures estimated from mean-squared displacement of the atoms.

| Resin | glass transition temperature (K) |
|---|---|
| **I** | 417.63 ± 47.69 |
| **II** | 463.62 ± 37.86 |
| **III** | 437.07 ± 49.55 |
| **IV** | 442.11 ± 34.97 |
| **V** | 544.53 ± 60.50 |
| **VI** | 517.54 ± 43.22 |

# References

(1) White, S. R.; Mather, P.; Smith, M. Characterization of the cure-state of DGEBA-DDS epoxy using ultrasonic, dynamic mechanical, and thermal probes. *Polym. Eng. Sci.* **2002**, *42*, 51–67.

(2) Alessi, S.; Caponetti, E.; Güven, O.; Akbulut, M.; Spadaro, G.; Spinella, A. Study of

the curing process of DGEBA epoxy resin through structural investigation. *Macromol. Chem. Phys.* **2015**, *216*, 538–546.

(3) Marks, M. J.; Snelgrove, R. V. Effect of conversion on the structure- property relationships of amine-cured epoxy thermosets. *ACS Appl. Mater. Interfaces* **2009**, *1*, 921–926.

(4) Li, C.; Medvedev, G. A.; Lee, E.-W.; Kim, J.; Caruthers, J. M.; Strachan, A. Molecular dynamics simulations and experimental studies of the thermomechanical response of an epoxy thermoset polymer. *Polymer* **2012**, *53*, 4222–4230.

(5) instruments, T. Frequency Dependence of Glass Transition Temperatures. `https://www.tainstruments.com/pdf/literature/TA423.pdf`, Last accessed on 28-03-2024.

(6) Humphrey, W.; Dalke, A.; Schulten, K. VMD – Visual Molecular Dynamics. *J. Mol. Graph.* **1996**, *14*, 33–38.

(7) Underwood, T.; Purton, J.; Manning, J.; Brukhno, A.; Stratford, K.; Düren, T.; Wilding, N.; Parker, S. dlmontepython: A Python library for automation and analysis of Monte Carlo molecular simulations. *arXiv preprint arXiv:2104.03822* **2021**,

(8) pymbar. `https://github.com/choderalab/pymbar`, Last accessed on 24-03-2024.

(9) Chodera, J. D. A simple method for automated equilibration detection in molecular simulations. *J. Chem. Theory Comput.* **2016**, *12*, 1799–1805.

(10) Coveney, P. V.; Wan, S. On the calculation of equilibrium thermodynamic properties from molecular dynamics. *Phys. Chem. Chem. Phys.* **2016**, *18*, 30236–30240.

(11) Wade, A. D.; Bhati, A. P.; Wan, S.; Coveney, P. V. Alchemical Free Energy Estimators and Molecular Dynamics Engines: Accuracy, Precision, and Reproducibility. *J. Chem. Theory Comput.* **2022**, *18*, 3972–3987.

(12) Siachouli, P.; Karadima, K. S.; Mavrantzas, V. G.; Pandis, S. N. The effect of functional groups on the glass transition temperature of atmospheric organic compounds: a molecular dynamics study. *Soft Matter* **2024**, *20*, 4783–4794.

(13) Vuković, F.; Swan, S. R.; Reyes, L. Q.; Varley, R. J.; Walsh, T. R. Beyond the ring flip: A molecular signature of the glass–rubber transition in tetrafunctional epoxy resins. *Polymer* **2020**, *206*, 122893.

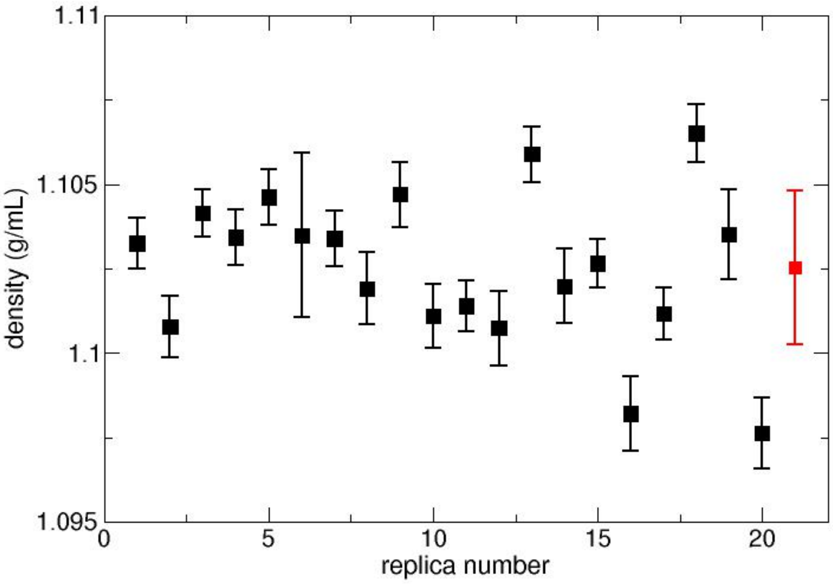


a)

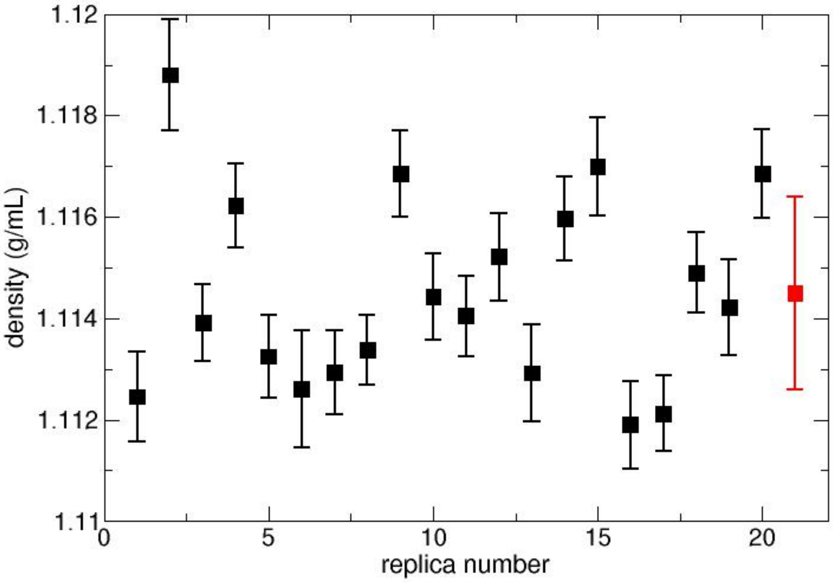


b)

Figure S13: Comparison of the standard deviation of densities computed using block averaging for each replica in the ensemble, compared to the standard deviation computed across the ensemble using the mean density of each replica. a) Resin model **II** at 300K using the Parallelhot protocol. Black points and error bars show the standard deviation computed using block averages for 7-12ns of simulation. The red point and error bar show the corresponding average and standard deviation are red. b) as in a) but computed using the stepwise_2ns protocol.

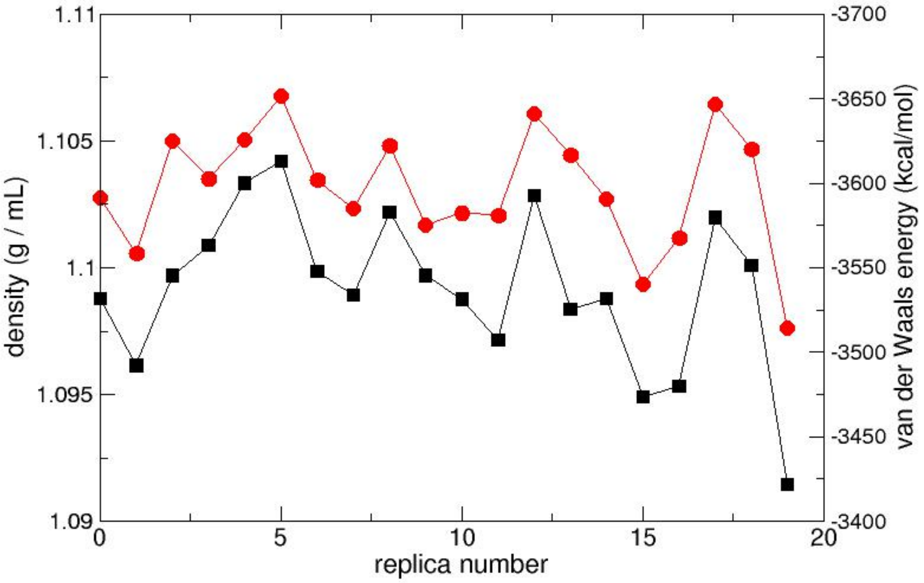


Figure S14: The average density (black lines, left y-axis) and the van der Waals energy (red line, right y-axis) for each replica within the ensemble for model **II** at 300K using the stepwise_2ns protocol. The correlation between the two values can clearly be seen, and is confirmed by the high Pearson correlation coefficients in Table S9.

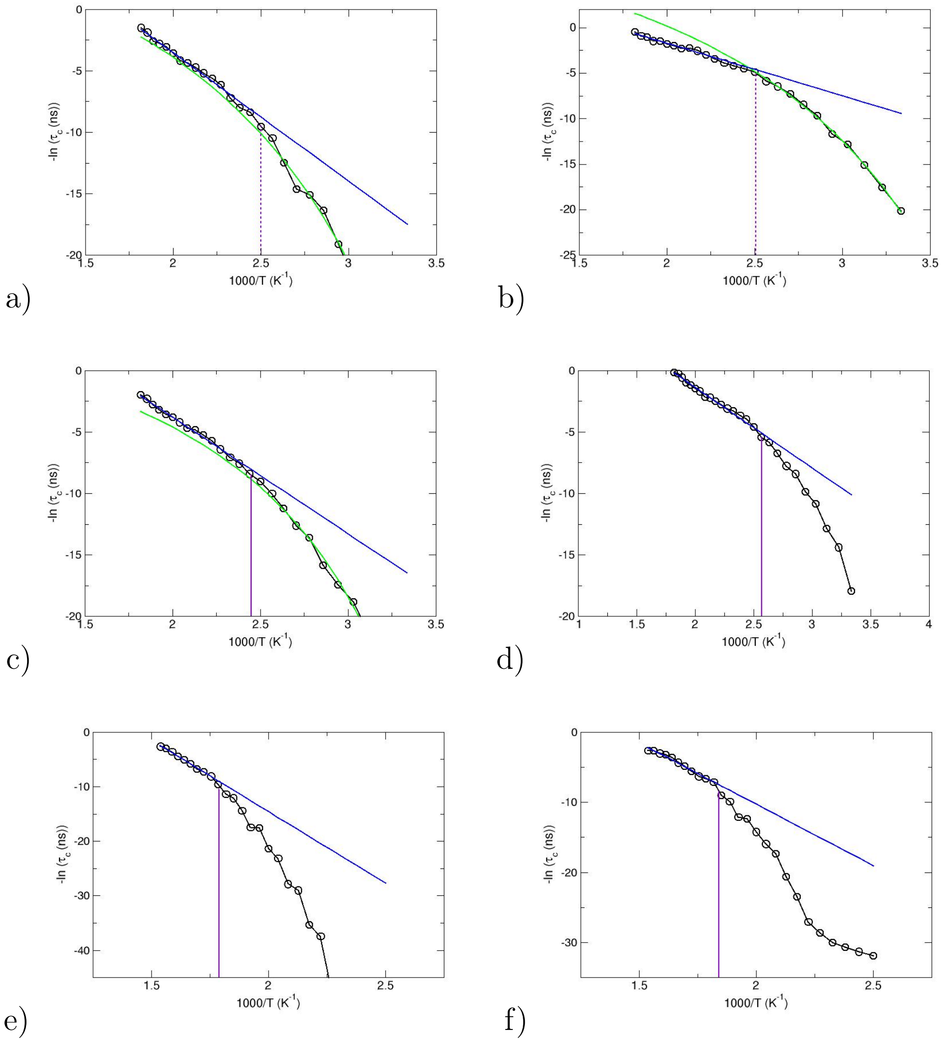


Figure S15: Relaxation times for the phenyl carbon-hydrogen bonds as a function of temperature (black circles). Fitting to Arrhenius behaviour is shown by blue lines, while fitting to VFT behaviour (where the fitting converged) is shown by green lines. The bond vectors are averaged over all bonds in the ensemble. The purple lines indicate the start of deviation from Arrhenius behaviour; these are converted to temperatures in Table S10. The models are: a) model **I**, b) **II**, c) **III**, d) **IV**, e) **V** and f) **VI**

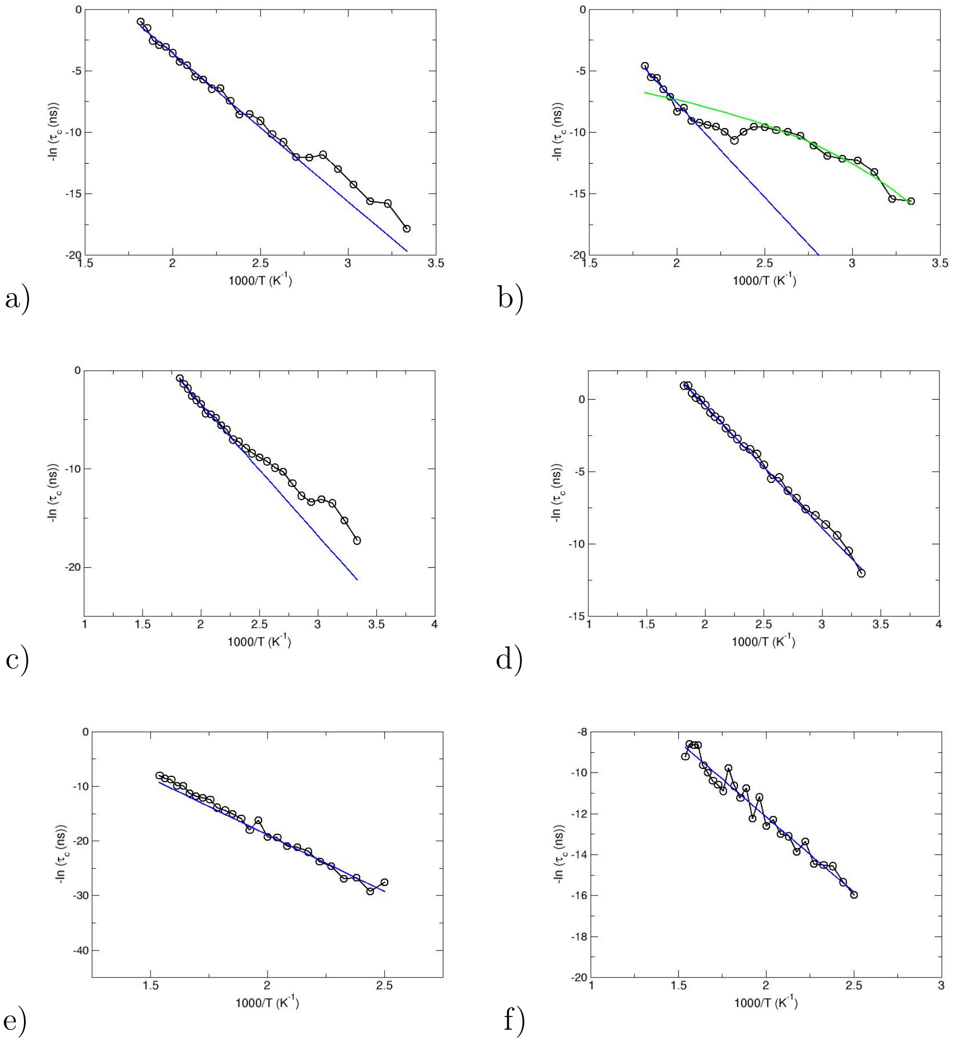


Figure S16: Relaxation times for the phenyl carbon-oxygen bonds as a function of temperature (black circles). Fitting to Arrhenius behaviour is shown by blue lines. The bond vectors are averaged over all bonds in the ensemble. We do not observe a transition to VFT behaviour, unlike for the phenyl C-H bonds. The models are: a) model **I**, b) **II**, c) **III**, d) **IV**, e) **V** and f) **VI**

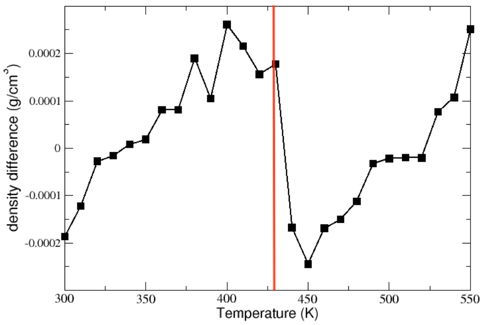


a)

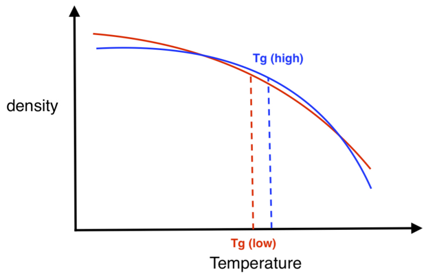


b)

Figure S17: a) The difference in average density at each temperature point between high $T_g$ and low $T_g$ density-temperature curves for Stepwise_density_2ns scenario for model **II**. For $T_g$ above the overall average $T_g$, the densities leading to a high $T_g$ are lower, while below 429K they are higher. Note, the overall average $T_g$ is 426K, indicated with a red line. In b) we show a sketch illustrating how this difference in density leads to different $T_g$.
